# Supplementary figures and images for: Galactosyl- and glucosylsphingosine induce lysosomal membrane permeabilization and cell death in cancer cells
Source: PLoS One. 2022 Nov 21;17(11):e0277058. doi: 10.1371/journal.pone.0277058 (PMC9678304; doi:10.1371/journal.pone.0277058)

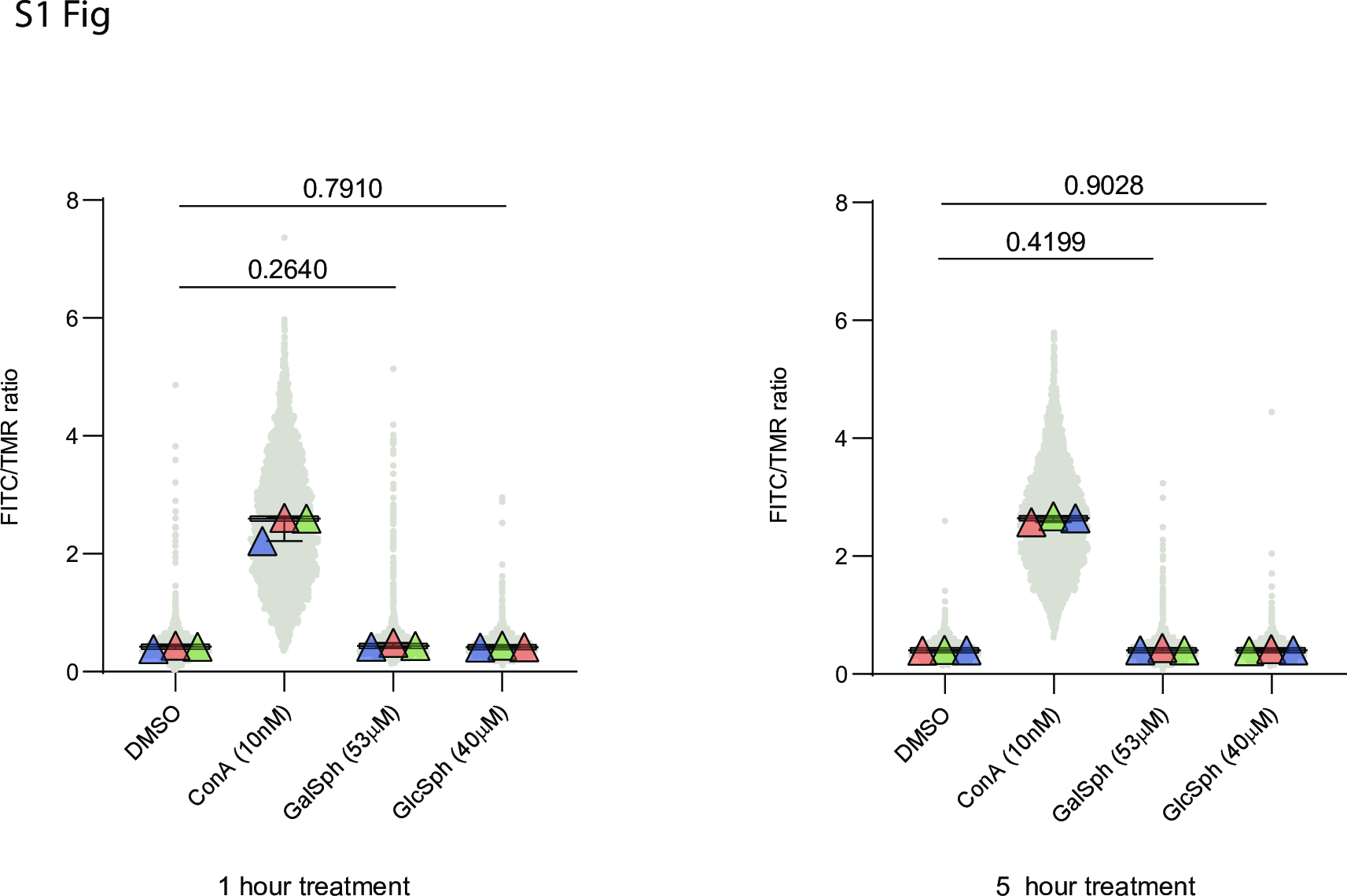

Supplement: S1 Fig — The graph indicates measured fluorescein (FITC) and pH-insensitive tetramethylrhodamine (TMR) intensity ratios of vesicles in MCF7 cells treated with vehicle, 10 nM concanamycin A, 53 μM GalSph, or 40 μM GlcSph for 1 or 5 hours. Small circles in light grey symbolizes all data points, large dark grey circles indicate technical replicates, and triangles specify biological replicates colorcoded as individual replicates. P-values calculated by unpaired student t-test are shown in the graph. Concanamycin A, a V-ATPase inhibitor, was used as a positive control for lysosomal neutralization. (TIF) [file pone.0277058.s001.tif]

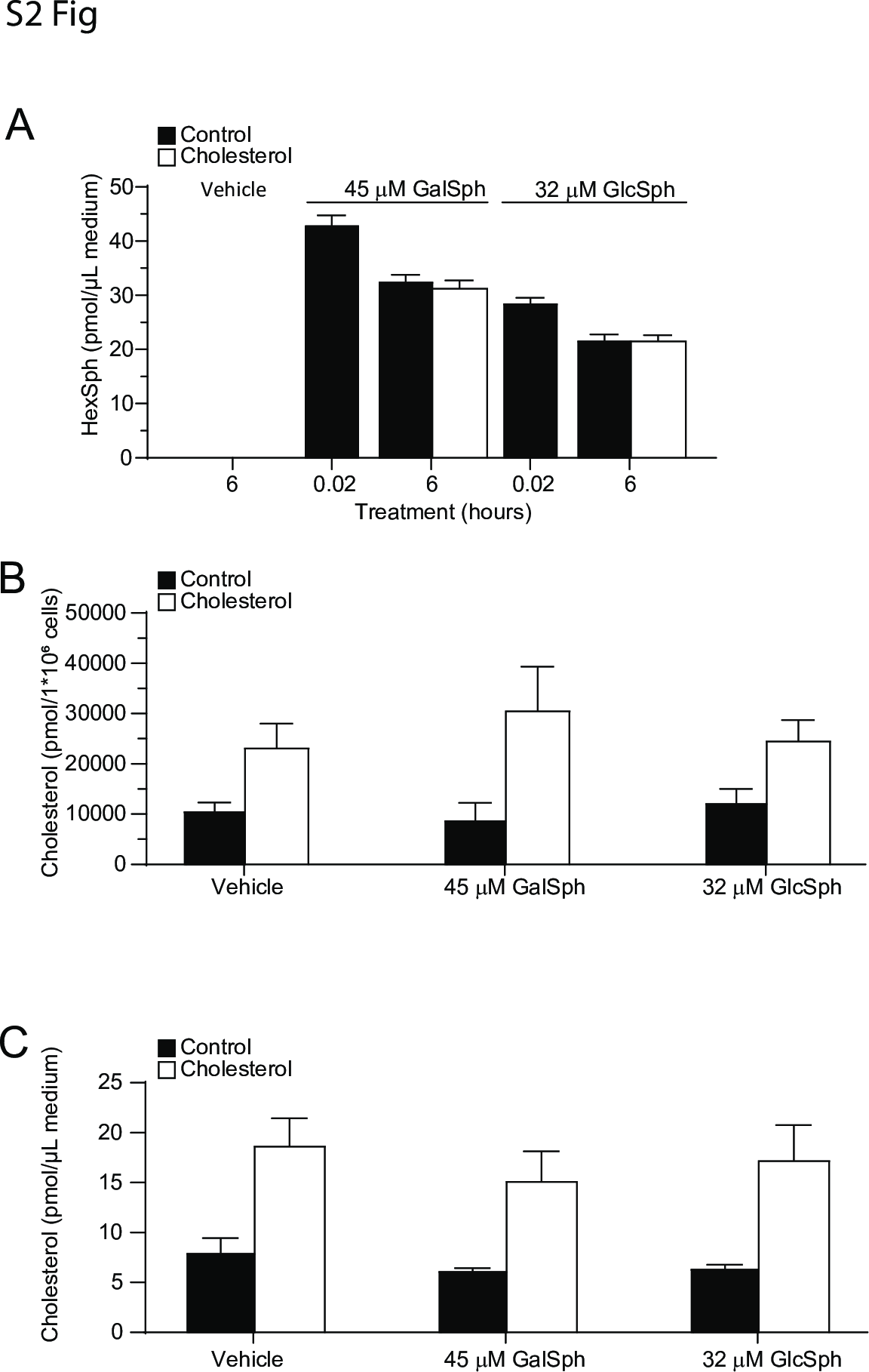

Supplement: S2 Fig — (A) Quantities of HexSph in cell culture medium of MCF7 cells treated as indicated. (B-C) Quantities of cholesterol in lysates (B) and cell culture medium (C) of MCF7 cells treated as indicated at the time point 6 hours after the addition of GalSph or GlcSph. Error bars, SD of three independent experiments. (TIF) [file pone.0277058.s002.tif]
