# Supplementary material for: Galactosyl- and glucosylsphingosine induce lysosomal membrane permeabilization and cell death in cancer cells
Source: PLoS One. 2022 Nov 21;17(11):e0277058. doi: 10.1371/journal.pone.0277058 (PMC9678304; doi:10.1371/journal.pone.0277058)
Supplement: S1 Table — (PDF) [file pone.0277058.s003.PDF]

S1 Table. Table of reagents and chemicals.

**Product**	**Source**	**Catalog number**
Paraformaldehyde (4%) in DPBS	Th Geyer	JM/J61899
Ammonium bicarbonate	VWR	40867
Ammonium chloride	Sigma-Aldrich	A0171
BSA	New England Biolabs	B9001S
Cholesterol	Sigma-Aldrich	C3045
Clarity Western ECL substrate	Bio-Rad	170-5061
cOmplete, Mini, EDTA-free protease inhibitor cocktail tablets	Roche	11697498001
DMEM	Thermos Fisher Scientific	31966-021
DMSO	VWR	VWRCN182
Dulbecco’s phosphate-buffered saline (DPBS)	Life technologies	14190-094
Ebastine	Cayman Chemical	15372
Ferrostatin-1	Abcam	ab146169
Forskolin	Sigma-Aldrich	93049
G418	Life Technologies	11811-031
Galactosylsphingosine	Avanti Polar Lipids	860537P
Glucosylsphingosine	Avanti Polar Lipids	860535P

Goat serum	Dako	X0907
Hoechst-33342	Sigma-Aldrich	B2261
Lipofectamine^{TM} RNAiMAX	Thermo Fisher Scientific	13778075
Methanol	Fluka	34966
Necrostatin-1	Sigma-Aldrich	N9037
Non-essential amino acids	Invitrogen	11140035
Opti-MEM	Life Technologies	31985-062
Penicillin and streptomycin	Thermo-Fisher	15070063
Phosphatase inhibitor cocktail tablets	Roche	04906837001
Prolong Gold Antifade mounting medium	Prolong Gold Antifade mounting medium	P36934
Propidium iodide	Sigma-Aldrich	P4864
Siramesine	H. Lundbeck A/S	Gift from Christine Volbracht
Sodium azide	Fluka	71289
TripLE^{TM} Express Enzyme (1X), no phenol red	Thermo Fisher Scientific	12604013
Z-VAD-FMK	Biomol	P-416
