# Supplementary material for: Galactosyl- and glucosylsphingosine induce lysosomal membrane permeabilization and cell death in cancer cells
Source: PLoS One. 2022 Nov 21;17(11):e0277058. doi: 10.1371/journal.pone.0277058 (PMC9678304; doi:10.1371/journal.pone.0277058)
Supplement: S2 Table — (PDF) [file pone.0277058.s004.PDF]

S2 Table. Table of antibodies.

**Product**	**Source**	**Catalog number**
Alpha-tubulin-HRP	Abcam	ab40742
Anti-mouse-HRP	Abcam	ab131368
Anti-rabbit-HRP	Cell Signalling Technology	7074
Caspase-7	Cell Signalling Technology	9492
Donkey anti-mouse IgG AlexaFluor-568	Thermo Fisher Scientific	A-10037
Donkey anti-rabbit IgG AlexaFluor-488	Thermo Fisher Scientific	A-21206
Galectin-3	Sigma-Aldrich	MABT51
Goat anti-rat IgG AlexaFluor-488	Thermo Fisher Scientific	A-11006
LAMP2	DSHB	H4B4-S
Nuclear Violet™ LCS1	AAT Bioquest	17543
P2RX4	Labome	APR-002
PARP	Cell Signalling Technology	9532
SiR-Tubulin	Cytoskeleton, Inc	CY-SC002
