# Supplementary material for: Galactosyl- and glucosylsphingosine induce lysosomal membrane permeabilization and cell death in cancer cells
Source: PLoS One. 2022 Nov 21;17(11):e0277058. doi: 10.1371/journal.pone.0277058 (PMC9678304; doi:10.1371/journal.pone.0277058)
Supplement: S3 Table — (PDF) [file pone.0277058.s005.PDF]

S3 Table. Table of siRNA.

**Product**	**Source**	**Catalog number**
P2RX4	Santa Cruz	sc-42569
