# Supplementary material for: Galactosyl- and glucosylsphingosine induce lysosomal membrane permeabilization and cell death in cancer cells
Source: PLoS One. 2022 Nov 21;17(11):e0277058. doi: 10.1371/journal.pone.0277058 (PMC9678304; doi:10.1371/journal.pone.0277058)
Supplement: S4 Table — (PDF) [file pone.0277058.s006.PDF]

S4 Table. Internal lipid standards.

Lipid class	Molecular Species formula	Source	ID	Amount added (pmol)
FA	FA 16:0-D4	TRC-Canada	P145502	24
DAG	DAG 12:0/12:0	Avanti	800812	8
TAG	TAG 17:0/17:0/17:0	Larodan	33-1700	24.39
PA and PA O-	PA 12:0/12:0	Avanti	840635	16.34
PC and PC O-	PC 12:0/12:0	Avanti	850335	20
PE and PE O-	PE 12:0/12:0	Avanti	850702	20
PG and PG O-	PG 12:0/12:0	Avanti	840435	11.03
BMP	BMP 14:0/14:0	Avanti	110857	12
PI and PI O-	PI 8:0/8:0	Avanti	850181	10.89
PS and PS O-	PS 12:0/12:0	Avanti	840038	6.63
CL	CL 14:0/14:0/14:0/14:0	Avanti	710332	20
LPA and LPA O-	LPA 17:0	Avanti	11067	17.64
LPC and LPC O-	LPC 12:0	Avanti	855475	16
LPE and LPE O-	LPE 13:0	Avanti	110696	17.36

LPG and LPG O-	LPG 17:1	Avanti	858127	10.46
LPI and LPI O-	LPI 13:0	Avanti	110716	11.28
LPS and LPS O-	LPS 17:1	Avanti	858141	14.72
Ceramide-1-phosphate (CerP)	CerP 18:1;2/12:0;0	Avanti	860531	16
Ceramide (Cer)	Cer 18:1;2/12:0;0	Avanti	860512	16
SHexCer	SHexCer 18:1;2/12:0;0	Avanti	860573	16
HexCer	HexCer 18:1;2/12:0;0	Avanti	860543	20
diHexCer	diHexCer 18:1;2/17:0;0	Avanti	860595	9.84
triHexCer	triHexCer18:1;2/17:0;0	Larodan	56-1061	12
GM3	GM3 18:1;2/18:0;0-D3	Larodan	71-1107	26
GM2	GM2 18:1;2/18:0;0-D3	Larodan	71-1200	26
GM1	GM1 18:1;2/18:0;0-D3	Larodan	71-1101	26
HexSph	GlcSph-$^{13}C_6$ 18:1;2	Larodan	78-4018	16
LSM	LSM 17:1;2	Avanti	110752	16

SM	SM 18:1;2/12:0;0	Avanti	860583	13.62
Cholesterol	Chol-D4	QMX	D-6359	196.25
CE	CE-D7 15:0	Avanti	700144	17.78

Avanti, Avanti Polar Lipids
