# Supplementary material for: Galactosyl- and glucosylsphingosine induce lysosomal membrane permeabilization and cell death in cancer cells
Source: PLoS One. 2022 Nov 21;17(11):e0277058. doi: 10.1371/journal.pone.0277058 (PMC9678304; doi:10.1371/journal.pone.0277058)
Supplement: S5 Table — (PDF) [file pone.0277058.s007.PDF]

S5 Table. Precursor ion, fragment ion and neutral loss for lipid identification

**Lipid class**	**Mode**	**MS1: Precursor ion**	**MS2: Fragment ion**	**MS2: Neutral loss**	**MS2: *m/z***	**MS2: Species specifics**
FA	NEG	$[M–H]^-$				
DAG	POS	$[M+NH_4]^+$		[Fatty acid – H + NH_4]		All
PA, PA O-, LPA, LPA O-	NEG	$[M–H]^-$	[Glycerophosphate – H – $H_2O]^-$		152.9958	All
		$[M–H]^-$	[Fatty acid – $H]^-$		*	PA, PA O-, LPA
		$[M–H]^-$	[Fatty acid O- – $H]^-$		*	PA O-
PC, PC O-, SM, LSM	POS	$[M+H]^+$	[Phosphorylcholine + $H]^+$		184.0733	All
LPC, LPC O-	NEG	$[M–H]^-$	[Fatty acid – $H]^-$		*	LPC
PE, PE O-, LPE, LPE O-	NEG	$[M–H]^-$	[Ethanolaminephosphate – H – $H_2O]^-$		196.038	All

		[M–H]$^-$	[Fatty acid – H]$^-$		*	PE, PE O-, LPE
		[M–H]$^-$	[Fatty acid O- – H]$^-$		*	PE O-
BMP/PG, LPG, LPG O-	NEG	[M–H]$^-$	[Glycerophosphate – H – H_2O]$^-$		152.9958	All
		[M–H]$^-$	[Fatty acid – H]$^-$		*	BMP/PG, LPG
PI, PI O-, LPI, LPI O-	NEG	[M–H]$^-$	[Glycerophosphate – H – H_2O]$^-$		152.9958	All
		[M–H]$^-$	[Inositolphosphate – H – H_2O]$^-$		241.0119	All
		[M–H]$^-$	[Fatty acid – H]$^-$		*	PI, PI O-, LPI
		[M–H]$^-$	[Fatty acid O- – H]$^-$		*	PI O-
PS, PS O–, LPS, LPS O–	NEG	[M–H]$^-$	[Glycerophosphate – H – H_2O]$^-$		152.9958	All
		[M–H]$^-$		[$C_3H_5NO_2$]		All

				Δ*m/z*: 87.032		
		[M–H]$^-$	[Fatty acid – H]$^-$		*	PS, PS O-, LPS
		[M–H]$^-$	[Fatty acid O- – H]$^-$		*	PS O-
CL	NEG	[M1–2H]^{2†}	[Fatty acid – H]$^-$		*	All
Cer, HexCer, diHexCer, triHexCer	POS	[M+H]$^+$	[LCB + H – H_2O]$^+$		*	All
		[M+H]$^+$	[LCB + H – $2H_2O$]$^+$		*	All
HexSph	POS	[M+H]$^+$	[LCB + H – $2H_2O$]$^+$		*	All
CerP	NEG	[M–H]$^-$	[Phosphoric acid – H – H_2O]$^-$		78.959	All
SHexCer	NEG	[M–H]$^-$	[HO_4S]$^-$		96.9601	All
GM3, GM2, GM1	NEG	[M–H]$^-$	[NeuAc – H]$^-$		290.0864	All

CE	POS	$[M+NH_4]^+$	$[Chol - NH_3 - H_2O]^+$		369.3516	All
Chol	POS (SIM/ tPRM)	$[M+NH_4]^+$	$[Chol - NH_3 - H_2O]^+$		369.3516	All

* Depends on species.

† M1 ion (first isotope) for identification of CL instead of M ion (mono isotope).

SIM: selected ion monitoring.

tPRM: targeted parallel reaction monitoring.
