# Supplementary material for: Galactosyl- and glucosylsphingosine induce lysosomal membrane permeabilization and cell death in cancer cells
Source: PLoS One. 2022 Nov 21;17(11):e0277058. doi: 10.1371/journal.pone.0277058 (PMC9678304; doi:10.1371/journal.pone.0277058)
Supplement: S6 Table — (PDF) [file pone.0277058.s008.PDF]

**S6 Table. Quantities (mol%) of lipid species identified in GalSph- and GlcSph-treated MCF7 cells and their lysosomes.**

| Experiment no. | 1 | 1 | 1 | 1 | 1 | 1 |
|---|---|---|---|---|---|---|
| Sample type | whole cell lysate | whole cell lysate | whole cell lysate | lysosomes | lysosomes | lysosomes |
| Treatment | control | GalSph | GlcSph | control | GalSph | GlcSph |
| CE 14:0 | 0,001605187 | 0,030136476 | 0,011651408 | 0 | 0,09453437 | 0,01709123 |
| CE 16:0 | 0,011622901 | 0,370901868 | 0,252503974 | 0 | 0,93390914 | 0,72517354 |
| CE 16:1 | 0 | 0,184842102 | 0,095004114 | 0 | 0,59792264 | 0,17731477 |
| CE 18:0 | 0 | 0,013900128 | 0,010408124 | 0 | 0,10937354 | 0,06683104 |
| CE 18:1 | 0,099684768 | 0,720874589 | 0,506650027 | 0 | 2,39157197 | 1,56867775 |
| CE 18:2 | 0 | 0,201929416 | 0,128307852 | 0 | 0,57961086 | 0,31397291 |
| CE 20:3 | 0 | 0,004777428 | 0,00249713 | 0 | 0,01956759 | 0 |
| CE 20:4 | 0 | 0,183771115 | 0,110178067 | 0 | 0,4060529 | 0,19870561 |
| CE 20:5 | 0 | 0,036155418 | 0,005026635 | 0 | 0,09885849 | 0,00500159 |
| CE 22:6 | 0 | 0,050116839 | 0,022586623 | 0 | 0,30146642 | 0,17289598 |
| Cer 32:1;2 | 0,005834391 | 0,004395548 | 0,005080633 | 0 | 0 | 0 |
| Cer 34:1;2 | 0,101565813 | 0,211224855 | 0,161541388 | 0,15138524 | 0,20669271 | 0,12423395 |
| Cer 34:2;2 | 0,025065088 | 0,020334738 | 0,0254922 | 0 | 0,01203524 | 0 |
| Cer 36:1;2 | 0 | 0,006978548 | 0,010605376 | 0 | 0,02307658 | 0 |
| Cer 36:2;2 | 0,002174698 | 0 | 0 | 0 | 0,0047602 | 0 |
| Cer 40:1;2 | 0,011813216 | 0,029420461 | 0,031423009 | 0 | 0 | 0 |
| Cer 40:2;2 | 0,006796641 | 0,019171813 | 0,023993081 | 0 | 0 | 0 |
| Cer 42:1;2 | 0,070548493 | 0,118207592 | 0,100081743 | 0 | 0,03907287 | 0,00177199 |
| Cer 42:2;2 | 0,164029008 | 0,304423898 | 0,317206261 | 0,00965722 | 0,18155986 | 0,10424297 |
| Cer 42:3;2 | 0,043602786 | 0,066223645 | 0,073507995 | 0 | 0,00932905 | 0 |
| Cer 44:1;2 | 0,015283317 | 0 | 0 | 0 | 0 | 0 |
| Cer 44:2;2 | 0,039947325 | 0 | 0,005982985 | 0 | 0 | 0 |
| Chol : | 23,67649661 | 23,12684083 | 25,8403404 | 48,5346366 | 42,4795079 | 46,0800499 |
| CL 62:2 | 0,012502944 | 0,390255245 | 0,227351705 | 0 | 0,60885649 | 0 |
| CL 64:4 | 0,045186713 | 0,01795004 | 0,054457029 | 0 | 0 | 0 |
| CL 66:2 | 0,024647719 | 0,021189771 | 0,034408532 | 0 | 0 | 0 |

| | | | | | | |
|---|---|---|---|---|---|---|
| CL 66:4 | 1,252329265 | 0,676305492 | 0 | 0 | 0 | 0 |
| CL 66:5 | 0,033459756 | 0,084747196 | 0,029333028 | 0 | 0,0104231 | 0 |
| CL 68:2 | 0,153287169 | 0,154173758 | 0,077358477 | 0 | 0 | 0 |
| CL 68:3 | 0,124255903 | 0,122333351 | 0,132545864 | 0 | 0 | 0 |
| CL 68:4 | 2,228348213 | 1,244616718 | 3,050523057 | 0 | 0,00208386 | 0 |
| CL 68:5 | 0,360088812 | 0,239562431 | 0,626058675 | 0 | 0 | 0 |
| CL 70:4 | 1,270058747 | 0,73838856 | 1,961014957 | 0 | 0 | 0 |
| CL 70:5 | 0,730709414 | 0,409549479 | 0,687162522 | 0 | 0 | 0 |
| CL 70:6 | 0,015706034 | 0,015032626 | 0,014541243 | 0 | 0 | 0 |
| CL 72:4 | 0,058566965 | 0,059865968 | 0,074176715 | 0 | 0 | 0 |
| CL 72:5 | 0,053214047 | 0,055387524 | 0,04432176 | 0 | 0 | 0 |
| CL 72:6 | 0,009460009 | 0,003471916 | 0 | 0 | 0 | 0 |
| DAG 28:0 | 0,007094603 | 0,00614063 | 0,005986043 | 0,05784519 | 0,02064991 | 0,04397467 |
| DAG 30:0 | 0,012039845 | 0,009702557 | 0,012577951 | 0 | 0 | 0 |
| DAG 30:1 | 0,021207011 | 0,023452286 | 0,02435536 | 0 | 0 | 0 |
| DAG 32:0 | 0,014101357 | 0,021520772 | 0,030693342 | 0 | 0 | 0 |
| DAG 32:1 | 0,14685313 | 0,15782568 | 0,170530738 | 0 | 0,03287974 | 0 |
| DAG 32:2 | 0,030363723 | 0,036722666 | 0,039300881 | 0 | 0 | 0,00160409 |
| DAG 34:1 | 0,259501573 | 0,264279676 | 0,267984283 | 0 | 0,04600047 | 0,00310425 |
| DAG 34:2 | 0,171367966 | 0,190607088 | 0,209312008 | 0,00398979 | 0,03366128 | 0,00477912 |
| DAG 34:3 | 0,007118294 | 0,00159883 | 0,003741715 | 0,01220313 | 0 | 0 |
| DAG 36:1 | 0,059202489 | 0,144404356 | 0,058473704 | 0 | 0 | 0 |
| DAG 36:2 | 0,261093988 | 0,267622674 | 0,281163323 | 0,00313506 | 0,04788371 | 0,01612898 |
| DAG 36:3 | 0,035744837 | 0,026340926 | 0,031879528 | 0 | 0 | 0 |
| DAG 36:4 | 0,023065266 | 0,009093057 | 0,011643467 | 0 | 0,00439193 | 0,00357123 |
| DAG 38:2 | 0,034216562 | 0,037243046 | 0,036946338 | 0 | 0 | 0 |
| DAG 38:3 | 0,007476623 | 0,03743175 | 0,006578625 | 0 | 0 | 0 |
| DAG 38:4 | 0,013603257 | 0,012850809 | 0,009459732 | 0,00111463 | 0 | 0 |
| DAG 38:5 | 0,015419252 | 0,012112756 | 0,017501413 | 0 | 0 | 0 |
| DAG 38:6 | 0,006969713 | 0,007353278 | 0,010558967 | 0 | 0 | 0 |
| DAG 40:5 | 0,005887977 | 0,004473174 | 0,002776255 | 0,00130859 | 0,00151012 | 0 |
| DAG 40:6 | 0,012741724 | 0,013760128 | 0,012855802 | 0 | 0 | 0 |

| | | | | | | |
|---|---|---|---|---|---|---|
| DAG 42:2 | 0,002046249 | 0,00206428 | 0,003301421 | 0 | 0 | 0 |
| diHexCer 42:2;2 | 0,011676584 | 0,009013736 | 0,009791788 | 0,00422907 | 0,01201359 | 0,0036979 |
| HexCer 32:1;2 | 0 | 0 | 0 | 0,00991798 | 0,0557415 | 0,03875103 |
| HexCer 34:0;2 | 0,00811434 | 0,002707151 | 0,005705774 | 0,10321616 | 0,05179019 | 0,06848345 |
| HexCer 34:1;2 | 0,167414817 | 0,2120053 | 0,190963797 | 0,67461684 | 0,75782641 | 0,69600211 |
| HexCer 34:2;2 | 0 | 0 | 0 | 0,00294896 | 0,01330277 | 0,00836454 |
| HexCer 36:1;2 | 0 | 0 | 0 | 0,00632249 | 0,08790435 | 0,06587763 |
| HexCer 36:2;2 | 0 | 0,00346584 | 0 | 0,00115413 | 0,00441538 | 0,00280462 |
| HexCer 40:1;2 | 0,063172922 | 0,045282601 | 0,056182044 | 0,13340262 | 0,16061145 | 0,12806995 |
| HexCer 40:2;2 | 0,008332723 | 0,004640052 | 0,00389001 | 0,02486414 | 0,05712634 | 0,04478214 |
| HexCer 42:1;2 | 0,173154692 | 0,158729251 | 0,166338303 | 0,34908012 | 0,3700402 | 0,28642036 |
| HexCer 42:2;2 | 0,352062612 | 0,383674048 | 0,399003913 | 0,79895402 | 0,9481673 | 0,71221921 |
| HexCer 42:3;2 | 0,005887044 | 0,009985221 | 0,009696946 | 0,01736982 | 0,06710359 | 0,0440006 |
| HexCer 44:1;2 | 0,045350756 | 0,031201443 | 0,034148273 | 0,08128644 | 0,10984481 | 0,05052489 |
| HexCer 44:2;2 | 0,126886678 | 0,113819019 | 0,127282513 | 0,27794792 | 0,26010228 | 0,19685519 |
| LPA 14:0 | 0 | 0 | 0 | 0 | 0 | 0 |
| LPA 16:0 | 0,01584154 | 0,024037791 | 0,031930463 | 0,10996382 | 0,0981213 | 0,16687599 |
| LPA 16:1 | 0 | 0 | 0 | 0,01611069 | 0,0190729 | 0,02010689 |
| LPA 18:0 | 0,003511319 | 0,003690264 | 0,010497607 | 0,01136644 | 0,0106684 | 0,02240196 |
| LPA 18:1 | 0,032195777 | 0,045986714 | 0,042375278 | 0,24145364 | 0,20505389 | 0,33772895 |
| LPA 18:2 | 0,001788249 | 0 | 0 | 0,00764237 | 0,00314127 | 0,00316094 |
| LPC 14:0 | 0,027249959 | 0,034589004 | 0,038607919 | 0,24040163 | 0,19239189 | 0,1777928 |
| LPC 16:0 | 0,177312477 | 0,232981716 | 0,251936905 | 2,11400025 | 1,59197511 | 1,4542144 |
| LPC 16:1 | 0,060123746 | 0,064570581 | 0,072197151 | 0,53721206 | 0,42833549 | 0,39391851 |
| LPC 18:0 | 0,045254532 | 0,084881608 | 0,085757114 | 0,34532934 | 0,33792607 | 0,33246665 |
| LPC 18:1 | 0,152522431 | 0,148441473 | 0,165484841 | 1,88926838 | 1,52759797 | 1,46987185 |
| LPC 18:3 | 0 | 0 | 0 | 0 | 0 | 0 |
| LPC 20:1 | 0,012909835 | 0,009987968 | 0,017287032 | 0,20847975 | 0,16140682 | 0,14953729 |
| LPC 20:3 | 0,00137543 | 0 | 0,001841933 | 0,01388673 | 0,04185326 | 0,04364063 |
| LPC 20:4 | 0,014505034 | 0,007293626 | 0,00977675 | 0,16888716 | 0,12727814 | 0,14223587 |
| LPC 22:5 | 0 | 0 | 0 | 0 | 0,02080456 | 0,02306682 |
| LPC 22:6 | 0 | 0 | 0 | 0 | 0,02658979 | 0,02786626 |

| | | | | | | |
|---|---|---|---|---|---|---|
| LPC O-16:0 | 0 | 0 | 0 | 0,00311572 | 0,00612359 | 0,00495223 |
| LPC O-16:1 | 0,001205297 | 0 | 0,002946133 | 0,02853181 | 0,04279023 | 0,02506741 |
| LPC O-18:0 | 0 | 0 | 0 | 0 | 0 | 0 |
| LPC O-18:1 | 0,001927913 | 0 | 0,003894934 | 0,01341592 | 0,0246924 | 0,01012648 |
| LPE 16:0 | 0 | 0,031110332 | 0 | 0,72370656 | 0 | 0 |
| LPE 16:1 | 0,025502059 | 0,021770203 | 0,025897181 | 0,07682953 | 0,09326473 | 0,08389281 |
| LPE 18:0 | 0,054091541 | 0,056177671 | 0,067529025 | 0,27814689 | 0,2118561 | 0,27294757 |
| LPE 18:1 | 0,152991511 | 0,143677833 | 0,168044003 | 0,74626736 | 0,8603568 | 0,80049359 |
| LPE 18:2 | 0,005701486 | 0 | 0,003519183 | 0,007546 | 0,0128423 | 0,00732513 |
| LPE 20:0 | 0 | 0 | 0 | 0 | 0 | 0 |
| LPE 20:1 | 0 | 0 | 0 | 0 | 0 | 0 |
| LPE 20:3 | 0 | 0 | 0 | 0 | 0 | 0 |
| LPE 20:4 | 0,025117258 | 0,016897511 | 0,02051996 | 0,06107145 | 0,06631241 | 0,05572766 |
| LPE 22:6 | 0,009772732 | 0,001786291 | 0,00620104 | 0,00905451 | 0,02394506 | 0,01411737 |
| LPE O-14:0 | 0 | 0 | 0 | 0 | 0 | 0 |
| LPE O-16:1 | 0 | 0 | 0 | 0,0137775 | 0,00974646 | 0,01079119 |
| LPE O-18:1 | 0,029217523 | 0,034328921 | 0,026914914 | 0,09545716 | 0,06292835 | 0,03684494 |
| LPE O-18:2 | 0 | 0 | 0 | 0,0014094 | 0,00410344 | 0,00153009 |
| LPE O-20:1 | 0 | 0 | 0 | 0 | 0 | 0 |
| LPG 14:0 | 0,002281463 | 0 | 0,001790983 | 0,02573211 | 0,01498318 | 0,01599615 |
| LPG 16:0 | 0 | 0 | 0,001619083 | 0,00643072 | 0,01242055 | 0,01207456 |
| LPG 16:1 | 0 | 0 | 0 | 0,00430476 | 0,01097649 | 0,00921693 |
| LPG 18:0 | 0 | 0 | 0 | 0,00246462 | 0,00501841 | 0,00852254 |
| LPG 18:1 | 0,015882673 | 0,016091809 | 0,020636295 | 0,30867366 | 0,20028274 | 0,25883684 |
| LPG 18:2 | 0 | 0 | 0 | 0,00431467 | 0,0038725 | 0,00312726 |
| LPG 22:6 | 0,002256602 | 0 | 0,001864313 | 0,13224339 | 0,07667093 | 0,10903996 |
| LPI 16:0 | 0,008902692 | 0,006407426 | 0,021320917 | 0,02038351 | 0,17818566 | 0,10239748 |
| LPI 16:1 | 0,019456293 | 0,007905697 | 0,008203783 | 0,061038 | 0,12735466 | 0,09644228 |
| LPI 18:0 | 0,131887614 | 0,163274305 | 0,194122299 | 1,17619221 | 0,9367193 | 1,20239091 |
| LPI 18:1 | 0,135239194 | 0,124196094 | 0,171458859 | 1,13443491 | 1,11121804 | 1,25426767 |
| LPS 18:0 | 0,01292272 | 0,01843335 | 0,022805667 | 0,08334018 | 0,12043071 | 0,13137775 |
| LPS 18:1 | 0,015631355 | 0,014926128 | 0,022812105 | 0,2413095 | 0,23889176 | 0,21952419 |

| | | | | | | |
|---|---|---|---|---|---|---|
| PA 30:1 | 0,002483682 | 0,003821419 | 0,005787618 | 0,02581462 | 0 | 0 |
| PA 32:1 | 0,029184299 | 0,046133538 | 0,049034087 | 0,00409457 | 0,01409702 | 0,00438026 |
| PA 32:2 | 0,007065348 | 0,010904787 | 0,015851234 | 0,00340509 | 0 | 0 |
| PA 34:1 | 0,036501091 | 0,056056479 | 0,053222089 | 0,00198639 | 0,00731017 | 0,00228156 |
| PA 34:2 | 0,040665268 | 0,05232486 | 0,060276343 | 0,00352553 | 0,00747553 | 0,00399107 |
| PA 36:2 | 0,042366532 | 0,054892957 | 0,05614512 | 0 | 0,00567532 | 0,00529139 |
| PA 36:3 | 0,018312425 | 0,008474031 | 0,014436123 | 0 | 0 | 0 |
| PA 36:4 | 0,004716871 | 0 | 0,00671325 | 0,05106556 | 0,00157103 | 0,09878263 |
| PA 38:3 | 0,006964538 | 0,004116325 | 0,004243233 | 0 | 0 | 0 |
| PA O-38:5 | 0,011119774 | 0,005639207 | 0 | 0,06260719 | 0,04038305 | 0,09684755 |
| PC 28:0 | 0,11479467 | 0,134130577 | 0,137436256 | 0,114146 | 0,10305283 | 0,0942112 |
| PC 30:0 | 0,469146422 | 0,507723941 | 0,512498373 | 0,51221427 | 0,4651632 | 0,46674744 |
| PC 30:1 | 1,083256884 | 1,039702502 | 1,078062059 | 0,63058878 | 0,5129496 | 0,43900263 |
| PC 32:0 | 0,387244702 | 0,365841388 | 0,376260134 | 0,51290941 | 0,52944119 | 0,60417788 |
| PC 32:1 | 7,516623453 | 6,688893024 | 7,075891061 | 3,10412335 | 2,89277696 | 2,42917774 |
| PC 32:2 | 1,867780864 | 1,680376847 | 1,798641176 | 0,77389989 | 0,66935934 | 0,5422771 |
| PC 34:1 | 11,03817679 | 9,092753593 | 9,843115754 | 3,10671277 | 3,60335805 | 3,18011751 |
| PC 34:2 | 8,664747415 | 7,237522335 | 7,82725911 | 2,35931112 | 2,42827931 | 1,81146759 |
| PC 34:3 | 0,394250719 | 0,324580412 | 0,342712501 | 0,13358272 | 0,13191446 | 0,09936508 |
| PC 34:4 | 0,374123421 | 0,297046994 | 0,248359556 | 0,12039102 | 0,08514768 | 0,07568778 |
| PC 36:1 | 0 | 0 | 0 | 0,16572722 | 0,46402579 | 0,42754967 |
| PC 36:2 | 11,56502546 | 9,164529564 | 10,08938384 | 1,75324682 | 2,37303318 | 1,70242037 |
| PC 36:3 | 1,103357841 | 0,856590091 | 0,943173742 | 0,23296608 | 0,29075303 | 0,19961754 |
| PC 36:4 | 0,999772005 | 0,766074915 | 0,729881015 | 0,25484341 | 0,27657764 | 0,21956131 |
| PC 36:5 | 0,620138566 | 0,471066171 | 0,436244031 | 0,11790927 | 0,15663529 | 0,09841558 |
| PC 38:1 | 0 | 0 | 0 | 0 | 0,01068902 | 0,00558941 |
| PC 38:2 | 1,245675291 | 1,013626041 | 1,119785629 | 0,12629535 | 0,21758478 | 0,15260609 |
| PC 38:3 | 0,211510927 | 0,190544478 | 0,200249045 | 0,00575896 | 0,10678589 | 0,07195885 |
| PC 38:4 | 0,256963805 | 0,215996875 | 0,238735539 | 0,04870335 | 0,15095525 | 0,10111126 |
| PC 38:5 | 1,033900524 | 0,747064405 | 0,739552841 | 0,1534225 | 0,248709 | 0,16284739 |
| PC 38:6 | 0,440375614 | 0,299847439 | 0,321857577 | 0 | 0 | 0 |
| PC 40:1 | 0,016450436 | 0,013426356 | 0,01769243 | 0 | 0 | 0 |

| | | | | | | |
|---|---|---|---|---|---|---|
| PC 40:2 | 0,140195594 | 0,110566866 | 0,114795641 | 0 | 0,01561228 | 0,00322441 |
| PC 40:3 | 0,048033975 | 0,040710204 | 0,046012965 | 0 | 0,00188548 | 0 |
| PC 40:4 | 0,059915316 | 0,04826404 | 0,053802822 | 0 | 0,00165935 | 0,00189105 |
| PC 40:5 | 0,225259481 | 0,182322117 | 0,181710106 | 0,00286932 | 0,07294081 | 0,03889801 |
| PC 40:6 | 0,247050233 | 0,190587711 | 0,206819498 | 0,00497707 | 0,10074876 | 0,04108873 |
| PC 40:7 | 0,352520236 | 0,212962472 | 0,24661536 | 0,00708466 | 0,02406248 | 0,01462847 |
| PC 40:8 | 0,050055057 | 0,057517661 | 0,03922941 | 0,03900993 | 0,18230299 | 0,03068659 |
| PC 42:1 | 0,00567334 | 0,006878896 | 0,008697847 | 0 | 0 | 0 |
| PC 42:2 | 0,112254617 | 0,09463578 | 0,102286613 | 0,00210529 | 0,01609024 | 0 |
| PC 42:3 | 0,027850085 | 0,020914319 | 0,025079669 | 0 | 0 | 0 |
| PC 42:4 | 0,008397361 | 0,003767825 | 0,006640968 | 0 | 0,0025186 | 0,00664124 |
| PC 42:5 | 0,026242253 | 0,016679044 | 0,019209316 | 0 | 0 | 0 |
| PC 42:6 | 0,233120676 | 0,168521893 | 0,188576527 | 0 | 0,01951224 | 0,00238244 |
| PC 42:7 | 0,047025207 | 0,029676847 | 0,033054531 | 0 | 0 | 0 |
| PC 42:8 | 0 | 0 | 0 | 0 | 0 | 0 |
| PC 42:9 | 0,066204674 | 0,053203087 | 0,050156149 | 0,00958474 | 0,01903925 | 0,0127217 |
| PC 44:2 | 0,069068046 | 0,066279693 | 0,067793132 | 0 | 0,00525234 | 0 |
| PC 44:3 | 0,017248714 | 0,013297849 | 0,015833544 | 0 | 0 | 0 |
| PC 44:6 | 0,044573388 | 0,028751388 | 0,033498328 | 0 | 0 | 0 |
| PC 44:7 | 0,00324347 | 0 | 0 | 0 | 0 | 0 |
| PC 44:9 | 0,084831609 | 0,057605961 | 0,061136213 | 0,00856756 | 0,01729804 | 0,01333326 |
| PC O-30:0 | 0 | 0 | 0 | 0 | 0,00904571 | 0,00845316 |
| PC O-30:1 | 0,001786681 | 0,003098001 | 0,002075172 | 0,00544576 | 0,01234456 | 0,01138726 |
| PC O-32:0 | 0 | 0 | 0 | 0,04441471 | 0,06863179 | 0,07868903 |
| PC O-32:1 | 0,002003051 | 0,006087336 | 0,003251306 | 0,06784558 | 0,10387746 | 0,10955929 |
| PC O-32:2 | 0,015975506 | 0,012854174 | 0,014938435 | 0,0073283 | 0,02342542 | 0,01185187 |
| PC O-34:0 | 0 | 0 | 0 | 0,00115708 | 0,00527675 | 0,00594634 |
| PC O-34:1 | 0 | 0,005451672 | 0 | 0,14308766 | 0,24659617 | 0,22977561 |
| PC O-34:2 | 0,002537064 | 0,009390336 | 0,010090475 | 0,05576817 | 0,1454554 | 0,12728126 |
| PC O-36:1 | 0 | 0 | 0 | 0 | 0,0203748 | 0,01312401 |
| PC O-36:2 | 0,013945231 | 0,020373966 | 0,0243093 | 0,00475455 | 0,0603912 | 0,03656274 |
| PC O-36:3 | 0,002689479 | 0,003122813 | 0,003187394 | 0 | 0,02510716 | 0,00774275 |

| | | | | | | |
|---|---|---|---|---|---|---|
| PC O-36:4 | 0,008086526 | 0 | 0,00346948 | 0,00132506 | 0,0230827 | 0,01884045 |
| PC O-36:5 | 0 | 0 | 0 | 0 | 0,02220851 | 0,00493018 |
| PC O-38:1 | 0,00175387 | 0,003292759 | 0,002837921 | 0 | 0,03481109 | 0,01094048 |
| PC O-38:2 | 0 | 0 | 0 | 0,00692132 | 0,00339196 | 0,00203711 |
| PC O-38:4 | 0 | 0 | 0,001372844 | 0 | 0,02158381 | 0,00485829 |
| PC O-38:6 | 0 | 0 | 0 | 0 | 0 | 0 |
| PC O-40:1 | 0,006577892 | 0,006226352 | 0,005568588 | 0,01029567 | 0,03922342 | 0,01071734 |
| PC O-40:5 | 0 | 0 | 0 | 0 | 0,01415628 | 0 |
| PC O-40:6 | 0 | 0 | 0 | 0 | 0,02889977 | 0,01739343 |
| PC O-40:7 | 0 | 0 | 0 | 0 | 0,02070537 | 0,00326026 |
| PC O-42:5 | 0 | 0,002651207 | 0 | 0 | 0,02474321 | 0 |
| PC O-42:6 | 0 | 0 | 0 | 0 | 0,00445546 | 0 |
| PC O-44:4 | 0,048323658 | 0,078100837 | 0,062325599 | 0,00179009 | 0,01746854 | 0 |
| PC O-44:5 | 0 | 0 | 0 | 0 | 0,00227569 | 0 |
| PE 32:1 | 0,199583488 | 0,443273741 | 0,234375984 | 0,01556779 | 0,1061496 | 0,14091097 |
| PE 34:1 | 0,596103824 | 1,556083176 | 0,645675969 | 0,16423788 | 0,22959182 | 0,31653161 |
| PE 34:2 | 0,853462204 | 1,744458198 | 1,002493163 | 0,35877037 | 0,49851129 | 0,53324307 |
| PE 34:3 | 0,036375275 | 0,076228374 | 0,043188162 | 0 | 0 | 0 |
| PE 36:1 | 0,333425167 | 0,861512861 | 0,309551691 | 0,01612619 | 0,04527856 | 0,09115214 |
| PE 36:2 | 2,082004423 | 4,876569084 | 2,213642514 | 0,5689257 | 0,90739674 | 1,03783192 |
| PE 36:3 | 0,197999793 | 0,400085421 | 0,206328858 | 0,00518697 | 0,05916248 | 0,06750508 |
| PE 36:4 | 0,169776082 | 0,341448371 | 0,17910031 | 0,00607822 | 0,04923913 | 0,05623623 |
| PE 36:5 | 0,070732281 | 0,116505065 | 0,074334083 | 0 | 0,00747613 | 0,00545952 |
| PE 38:2 | 0,130526069 | 0,342627484 | 0,136530657 | 0,00169814 | 0,02321126 | 0,03032327 |
| PE 38:3 | 0,053255456 | 0,11475658 | 0,046099503 | 0 | 0 | 0 |
| PE 38:4 | 0,342204717 | 0,775351923 | 0,327414718 | 0 | 0,04907842 | 0,04199128 |
| PE 38:5 | 0,526438768 | 1,045098197 | 0,558989103 | 0,05096469 | 0,19662939 | 0,16982 |
| PE 38:6 | 0,167503047 | 0,275185763 | 0,154683255 | 0 | 0,01139083 | 0,00512324 |
| PE 40:2 | 0,00119499 | 0,024946134 | 0 | 0 | 0 | 0 |
| PE 40:5 | 0 | 0,130915276 | 0,027205609 | 0 | 0 | 0 |
| PE 40:6 | 0,172629059 | 0,393230776 | 0,166940113 | 0 | 0 | 0 |
| PE 40:7 | 0,148162524 | 0,243828844 | 0,117083619 | 0 | 0,00556618 | 0 |

| | | | | | | |
|---|---|---|---|---|---|---|
| PG 32:1 | 0,004386518 | 0,009471424 | 0,010466808 | 0,09883811 | 0,07223042 | 0,10243904 |
| PG 32:2 | 0 | 0 | 0 | 0,04328809 | 0,05365196 | 0,06403958 |
| PG 34:1 | 0,081358656 | 0,103757128 | 0,089617857 | 0,25751944 | 0,19042034 | 0,31123686 |
| PG 34:2 | 0,036288984 | 0,040517553 | 0,040761237 | 0,98613026 | 0,74063791 | 0,86895514 |
| PG 34:3 | 0 | 0 | 0 | 0,08425643 | 0,06899385 | 0,08058177 |
| PG 36:2 | 0,137958033 | 0,133078726 | 0,131927543 | 3,30496085 | 2,52221537 | 3,09634885 |
| PG 36:3 | 0,015862485 | 0,015625183 | 0,014074776 | 0,50268468 | 0,38592015 | 0,44723682 |
| PG 36:4 | 0,001155306 | 0 | 0 | 0,13915732 | 0,11141637 | 0,11208566 |
| PG 36:5 | 0 | 0 | 0 | 0,01664345 | 0,02995483 | 0,02261105 |
| PG 38:3 | 0,007831911 | 0,003739513 | 0,006211506 | 0,18524679 | 0,17112809 | 0,18122708 |
| PG 38:4 | 0,01568247 | 0,012257909 | 0,015083765 | 0,44120098 | 0,40093247 | 0,38271273 |
| PG 38:5 | 0,00322108 | 0 | 0,001622203 | 0,2357662 | 0,1954319 | 0,18244386 |
| PG 38:6 | 0,003928927 | 0,00247768 | 0,003341093 | 0,19329757 | 0,14740794 | 0,1662549 |
| PG 38:7 | 0,004435365 | 0 | 0,003100742 | 0,31501773 | 0,22424899 | 0,22768891 |
| PG 40:5 | 0 | 0 | 0 | 0,07441472 | 0,07802723 | 0,0689838 |
| PG 40:6 | 0 | 0 | 0 | 0,20579448 | 0,18001762 | 0,1420866 |
| PG 40:7 | 0,085668123 | 0,0674692 | 0,066839316 | 2,5078079 | 1,76550145 | 1,73667301 |
| PG 40:8 | 0,002425023 | 0 | 0 | 0,18840987 | 0,12918405 | 0,1308721 |
| PG 42:10 | 0 | 0 | 0 | 0,10122083 | 0,07680855 | 0,05798101 |
| PG 42:7 | 0 | 0 | 0 | 0,02731247 | 0,03773752 | 0,02308471 |
| PG 42:8 | 0 | 0 | 0 | 0,08197632 | 0,07173873 | 0,0641445 |
| PG 42:9 | 0,003038592 | 0 | 0 | 0,20206145 | 0,16611283 | 0,14440812 |
| PG 44:10 | 0 | 0 | 0 | 0,00884311 | 0,02163248 | 0,00529576 |
| PG 44:11 | 0 | 0 | 0 | 0,10473887 | 0,06751957 | 0,0469769 |
| PG 44:12 | 0,023045749 | 0,014645833 | 0,015893509 | 0,73560962 | 0,4423874 | 0,40282599 |
| PI 30:0 | 0,001139858 | 0,005857504 | 0,007358438 | 0 | 0 | 0 |
| PI 30:1 | 0 | 0,003220619 | 0,004765257 | 0,00213117 | 0 | 0,00147435 |
| PI 32:0 | 0,034618802 | 0,077234164 | 0,059369677 | 0,02382605 | 0,04693362 | 0,12407068 |
| PI 32:1 | 0,160010341 | 0,229452353 | 0,230632575 | 0,07960519 | 0,12993701 | 0,18883342 |
| PI 34:1 | 0,901926162 | 1,098855682 | 0,949855581 | 0,65243625 | 0,57609355 | 1,06421807 |
| PI 34:2 | 0,339148868 | 0,489098512 | 0,518852258 | 0,18626112 | 0,25204691 | 0,34713122 |
| PI 36:1 | 0,914032931 | 0,968911091 | 0,69291404 | 0,61907936 | 0,39923422 | 0,85611324 |

| | | | | | | |
|---|---|---|---|---|---|---|
| PI 36:2 | 0,75111884 | 0,96261351 | 0,873885196 | 0,32117811 | 0,4197034 | 0,63801963 |
| PI 36:3 | 0,057934193 | 0,073772792 | 0,072577992 | 0 | 0,01151891 | 0,03203904 |
| PI 36:4 | 0,056334451 | 0,040628937 | 0,04658522 | 0,03464384 | 0,06616481 | 0,04620963 |
| PI 36:5 | 0,014316676 | 0,004964865 | 0,018612927 | 0 | 0,00374024 | 0,00192851 |
| PI 38:1 | 0,063534065 | 0,082226484 | 0,050246485 | 0,00542235 | 0,01120303 | 0,05301652 |
| PI 38:2 | 0,10952268 | 0,131205737 | 0,099658942 | 0,008386 | 0,02857771 | 0,06356601 |
| PI 38:3 | 0,105042021 | 0,104280793 | 0,085000226 | 0 | 0,01342257 | 0,04612291 |
| PI 38:4 | 0,131957263 | 0,107649061 | 0,104743119 | 0,01965697 | 0,06000579 | 0,09199609 |
| PI 38:5 | 0,088642292 | 0,089310992 | 0,093079904 | 0,00589275 | 0,03857834 | 0,02420077 |
| PI 38:6 | 0,075751938 | 0,097719132 | 0,098005079 | 0,00420069 | 0,0398647 | 0,02182722 |
| PI 40:3 | 0,066136134 | 0,061572393 | 0,066221923 | 0,50568105 | 0,12403764 | 0,38643943 |
| PI 40:4 | 0,01937406 | 0,020896747 | 0,01261928 | 0,01326069 | 0,00287939 | 0,03381416 |
| PI 40:5 | 0,046525744 | 0,052171895 | 0,039915583 | 0 | 0 | 0 |
| PI 40:6 | 0,139869506 | 0,146251707 | 0,129539244 | 0,00136651 | 0,03674869 | 0,04277588 |
| PI 40:7 | 0,058435482 | 0,087584 | 0,086577751 | 0,00165054 | 0,01401961 | 0,01125787 |
| PI 42:5 | 0,013006173 | 0,012811529 | 0,006298379 | 0 | 0 | 0 |
| PI 42:6 | 0,003682372 | 0,005836983 | 0,00427615 | 0 | 0 | 0 |
| PS 32:1 | 0,029124673 | 0,073984757 | 0,056839293 | 0,00317109 | 0,0828471 | 0,08891388 |
| PS 34:1 | 0,145534376 | 0,30096706 | 0,225657159 | 0 | 0,20521671 | 0,29420068 |
| PS 34:2 | 0,042082833 | 0,092106222 | 0,077452859 | 0 | 0,08587917 | 0,10428563 |
| PS 36:1 | 0,181178538 | 0,300309234 | 0,203014822 | 0,12562637 | 0,18840528 | 0,2988315 |
| PS 36:2 | 0,144572306 | 0,286180068 | 0,222029387 | 0 | 0,17301484 | 0,22102576 |
| PS 36:4 | 0,016253288 | 0,019945856 | 0,020034768 | 0 | 0,02108866 | 0,01397373 |
| PS 38:2 | 0,038838402 | 0,056015433 | 0,042475017 | 0,00790748 | 0,01732558 | 0,02478694 |
| PS 38:4 | 0,030125871 | 0,033861941 | 0,034121078 | 0 | 0,03506868 | 0,02641342 |
| PS 40:2 | 0,003815364 | 0,010630754 | 0,004733755 | 0 | 0 | 0 |
| PS 40:6 | 0 | 0 | 0 | 0 | 0 | 0 |
| PS 40:7 | 0,00655613 | 0,020415286 | 0,019082878 | 0 | 0,00554853 | 0,00408265 |
| PS 42:7 | 0,002002789 | 0 | 0 | 0 | 0 | 0 |
| SM 32:0;2 | 0,011832801 | 0,007152029 | 0,008447435 | 0,03452759 | 0,02449711 | 0,01774256 |
| SM 32:1;2 | 0,08991954 | 0,09124285 | 0,094224192 | 0,28671457 | 0,22415348 | 0,22217294 |
| SM 32:2;2 | 0 | 0 | 0 | 0,00226357 | 0,02026633 | 0,00624346 |

| | | | | | | |
|---|---|---|---|---|---|---|
| SM 34:0;2 | 0,339047398 | 0,285088744 | 0,305033854 | 0,90854922 | 0,52726266 | 0,60447278 |
| SM 34:1;2 | 1,38390524 | 1,524748539 | 1,511602237 | 4,36042566 | 3,60106699 | 3,8876441 |
| SM 34:2;2 | 0,1431097 | 0,141526243 | 0,141868585 | 0,46051555 | 0,38825649 | 0,35855802 |
| SM 36:0;2 | 0,002458352 | 0 | 0 | 0,00954295 | 0,00754736 | 0,00797324 |
| SM 36:1;2 | 0,049555329 | 0,071596005 | 0,070976645 | 0,20236502 | 0,2764165 | 0,34911029 |
| SM 36:2;2 | 0,008006855 | 0,015343236 | 0,020030386 | 0,03653503 | 0,0964645 | 0,1019898 |
| SM 38:1;2 | 0 | 0 | 0,002360943 | 0,05468147 | 0,1175814 | 0,13833791 |
| SM 38:2;2 | 0 | 0 | 0 | 0 | 0,01726351 | 0,01054877 |
| SM 40:0;2 | 0,002232466 | 0,001818966 | 0,002980811 | 0,00171779 | 0,0096308 | 0,00615599 |
| SM 40:1;2 | 0,070017709 | 0,109409388 | 0,112302602 | 0,20773862 | 0,37357387 | 0,39993312 |
| SM 40:2;2 | 0,068427396 | 0,069305173 | 0,078110365 | 0,12857095 | 0,22453104 | 0,2133048 |
| SM 42:1;2 | 0,096058279 | 0,101996291 | 0,108903947 | 0,12459398 | 0,21581775 | 0,17384652 |
| SM 42:2;2 | 0,717233705 | 0,67481742 | 0,750739439 | 0,95710001 | 1,22275511 | 1,11752165 |
| SM 42:3;2 | 0,111506105 | 0,107087893 | 0,118359039 | 0,10839582 | 0,20092067 | 0,16041799 |
| SM 44:2;2 | 0,082384025 | 0,069657282 | 0,075624157 | 0,06299302 | 0,08633761 | 0,0609036 |

| Experiment no. | 2 | 2 | 2 | 2 | 2 | 2 |
|---|---|---|---|---|---|---|
| Sample type | whole cell lysate | whole cell lysate | whole cell lysate | lysosomes | lysosomes | lysosomes |
| Treatment | control | GalSph | GlcSph | control | GalSph | GlcSph |
| CE 14:0 | 0 | 0,050745139 | 0,020220688 | 0 | 0,04241148 | 0,07935262 |
| CE 16:0 | 0,066929869 | 0,516753442 | 0,457373554 | 0,09957977 | 0,56624502 | 1,0348473 |
| CE 16:1 | 0 | 0,23540959 | 0,164203758 | 0 | 0,2559392 | 0,46842994 |
| CE 18:0 | 0 | 0,031405971 | 0,017611627 | 0 | 0,02815205 | 0,09472532 |
| CE 18:1 | 0,152617586 | 0,98909516 | 0,850814903 | 0,26538291 | 1,49847028 | 2,76887327 |
| CE 18:2 | 0,014655795 | 0,28499739 | 0,220196303 | 0 | 0,40879255 | 0,58054577 |
| CE 20:3 | 0 | 0,010214664 | 0,002604806 | 0 | 0,00926643 | 0,00430616 |
| CE 20:4 | 0,01310554 | 0,257000872 | 0,179000716 | 0,0071399 | 0,33545786 | 0,36370633 |
| CE 20:5 | 0 | 0,048713096 | 0,020473581 | 0,00186178 | 0,05337592 | 0,02027328 |
| CE 22:6 | 0 | 0,075217079 | 0,049491957 | 0 | 0,1842961 | 0,30796356 |
| Cer 32:1;2 | 0 | 0 | 0 | 0 | 0,02174097 | 0 |

| | | | | | | |
|---|---|---|---|---|---|---|
| Cer 34:1;2 | 0,1121757 | 0,156775682 | 0,110503314 | 0 | 0,33621135 | 0,07074068 |
| Cer 34:2;2 | 0,016817397 | 0,006912323 | 0,009708874 | 0 | 0,00783702 | 0,00399864 |
| Cer 36:1;2 | 0 | 0 | 0 | 0 | 0,07691145 | 0 |
| Cer 36:2;2 | 0 | 0 | 0 | 0 | 0,00291082 | 0 |
| Cer 40:1;2 | 0,00927927 | 0,026577667 | 0,031612617 | 0 | 0,02020607 | 0 |
| Cer 40:2;2 | 0,004217457 | 0,004025455 | 0,00659155 | 0 | 0,00336732 | 0 |
| Cer 42:1;2 | 0,054154655 | 0,101094669 | 0,121693057 | 0 | 0,01208941 | 0 |
| Cer 42:2;2 | 0,169932364 | 0,272483186 | 0,274478765 | 0,03065998 | 0,1829675 | 0,10582715 |
| Cer 42:3;2 | 0,031318921 | 0,045492753 | 0,041926674 | 0 | 0,00587465 | 0 |
| Cer 44:1;2 | 0 | 0 | 0 | 0 | 0 | 0 |
| Cer 44:2;2 | 0,025002514 | 0,019724147 | 0 | 0,00133225 | 0,00334746 | 0 |
| Chol : | 22,69459271 | 24,02933056 | 24,64522765 | 43,0216851 | 41,075344 | 47,0379766 |
| CL 62:2 | 0,276477991 | 0,120184326 | 1,702528645 | 0,02424895 | 0,1068596 | 0 |
| CL 64:4 | 0,019978779 | 0 | 0 | 0 | 0 | 0 |
| CL 66:2 | 0,028821388 | 0,02101447 | 0,003426417 | 0 | 0 | 0 |
| CL 66:4 | 0,499938181 | 0,259896081 | 0,154239915 | 0 | 0,01665233 | 0 |
| CL 66:5 | 0,024920328 | 0,008508112 | 0,110130552 | 0 | 0 | 0,01373339 |
| CL 68:2 | 0,264747438 | 0,212513053 | 0,107971561 | 0 | 0,00633884 | 0 |
| CL 68:3 | 0,138384513 | 0 | 0,238452991 | 0 | 0 | 0 |
| CL 68:4 | 1,319965846 | 1,174941626 | 1,159531554 | 0 | 0,04959016 | 0 |
| CL 68:5 | 0,200435087 | 0,121170783 | 0,097172346 | 0 | 0,00621322 | 0 |
| CL 70:4 | 0,880809916 | 0,893925931 | 0,864108244 | 0,00548714 | 0,02315845 | 0 |
| CL 70:5 | 0,582672709 | 0,254912298 | 0,338317421 | 0 | 0,01586229 | 0 |
| CL 70:6 | 0,029399601 | 0,007824827 | 0 | 0 | 0 | 0 |
| CL 72:4 | 0,058591949 | 0,05356754 | 0,067879915 | 0 | 0 | 0 |
| CL 72:5 | 0,090164031 | 0 | 0,045976609 | 0 | 0 | 0 |
| CL 72:6 | 0,034944168 | 0,009170553 | 0 | 0 | 0 | 0 |
| DAG 28:0 | 0,004144429 | 0,012269534 | 0,009234399 | 0 | 0,02896808 | 0,03905514 |
| DAG 30:0 | 0,008020782 | 0,017343759 | 0,006437525 | 0 | 0,0093156 | 0 |
| DAG 30:1 | 0,010998118 | 0,013660937 | 0,009128975 | 0,01477669 | 0 | 0 |
| DAG 32:0 | 0,008191816 | 0,023735857 | 0,014455484 | 0 | 0,02770874 | 0 |
| DAG 32:1 | 0,105541926 | 0,114236805 | 0,11070355 | 0 | 0,02188149 | 0,02120939 |

| | | | | | | |
|---|---|---|---|---|---|---|
| DAG 32:2 | 0,012477405 | 0,017855743 | 0,021165093 | 0 | 0 | 0 |
| DAG 34:1 | 0,22474338 | 0,229934668 | 0,184843582 | 0,00868898 | 0,0912706 | 0,04535343 |
| DAG 34:2 | 0,099455443 | 0,113136675 | 0,108542073 | 0,00207548 | 0,09876219 | 0,01595494 |
| DAG 34:3 | 0,009019019 | 0 | 0 | 0 | 0,0050303 | 0 |
| DAG 36:1 | 0,061920828 | 0,18172655 | 0,030714803 | 0 | 0,03546208 | 0 |
| DAG 36:2 | 0,191870714 | 0,19918026 | 0,179853038 | 0,00165891 | 0,23086238 | 0,02206547 |
| DAG 36:3 | 0,027142795 | 0,02133482 | 0,017257551 | 0 | 0,00217202 | 0 |
| DAG 36:4 | 0,016144587 | 0,009197402 | 0,007276457 | 0,01452795 | 0,00686644 | 0,00779218 |
| DAG 38:2 | 0,029309178 | 0,098915693 | 0,017752454 | 0 | 0,13294325 | 0 |
| DAG 38:3 | 0,002415716 | 0,018787482 | 0 | 0 | 0,01043236 | 0 |
| DAG 38:4 | 0,017138932 | 0,012617851 | 0,008022045 | 0,00694687 | 0,00228499 | 0 |
| DAG 38:5 | 0,018237675 | 0,01123107 | 0,009507648 | 0 | 0 | 0 |
| DAG 38:6 | 0,005627155 | 0,0085973 | 0,005838393 | 0 | 0 | 0 |
| DAG 40:5 | 0,002085737 | 0,00318404 | 0,003700329 | 0 | 0 | 0 |
| DAG 40:6 | 0,010263447 | 0,013545131 | 0,011334131 | 0 | 0 | 0 |
| DAG 42:2 | 0,001177154 | 0,001945575 | 0 | 0 | 0 | 0 |
| diHexCer 42:2;2 | 0,01230462 | 0,011146601 | 0 | 0,00194373 | 0,01601179 | 0,00776396 |
| HexCer 32:1;2 | 0 | 0 | 0 | 0,0217854 | 0,01716453 | 0,00507666 |
| HexCer 34:0;2 | 0 | 0 | 0 | 0 | 0,00230773 | 0 |
| HexCer 34:1;2 | 0,169996144 | 0,236385831 | 0,181321263 | 0,23962729 | 0,66043938 | 0,35807367 |
| HexCer 34:2;2 | 0 | 0 | 0 | 0,00143024 | 0,00976589 | 0 |
| HexCer 36:1;2 | 0 | 0,009637145 | 0 | 0,05523779 | 0,06528035 | 0,04057264 |
| HexCer 36:2;2 | 0 | 0,003363955 | 0 | 0 | 0,01764506 | 0 |
| HexCer 40:1;2 | 0,091528099 | 0,107967927 | 0,08095734 | 0,12513843 | 0,22634692 | 0,18412493 |
| HexCer 40:2;2 | 0 | 0,001941945 | 0,001822418 | 0,00630411 | 0,03651903 | 0,00509145 |
| HexCer 42:1;2 | 0,257071991 | 0,255112456 | 0,280674232 | 0,3254676 | 0,42231574 | 0,42817202 |
| HexCer 42:2;2 | 0,410947889 | 0,401331708 | 0,367793813 | 0,42449357 | 0,9623234 | 0,70328852 |
| HexCer 42:3;2 | 0,002650026 | 0,005242979 | 0,001475709 | 0 | 0,05211007 | 0,00850179 |
| HexCer 44:1;2 | 0,105190157 | 0,080272145 | 0,107463451 | 0,13070757 | 0,13623791 | 0,15884317 |
| HexCer 44:2;2 | 0,25818722 | 0,219929005 | 0,201329719 | 0,28098119 | 0,3970006 | 0,3355853 |
| LPA 14:0 | 0 | 0 | 0 | 0 | 0 | 0 |
| LPA 16:0 | 0,011571666 | 0,018026321 | 0,025582367 | 0,10349973 | 0,05298469 | 0,06308274 |

| | | | | | | |
|---|---|---|---|---|---|---|
| LPA 16:1 | 0,00385345 | 0,002032129 | 0,004645552 | 0,00534527 | 0,00455098 | 0,00602329 |
| LPA 18:0 | 0,003731076 | 0,005945221 | 0,00836328 | 0,01830779 | 0,01975573 | 0,01938481 |
| LPA 18:1 | 0,038152272 | 0,058127337 | 0,079941859 | 0,17815677 | 0,13525628 | 0,16703474 |
| LPA 18:2 | 0 | 0 | 0 | 0,00161984 | 0 | 0 |
| LPC 14:0 | 0,033978934 | 0,061062985 | 0,070853382 | 0,15174532 | 0,09704117 | 0,13858974 |
| LPC 16:0 | 0,184496017 | 0,374325307 | 0,407318643 | 1,38981124 | 0,87238411 | 1,2529931 |
| LPC 16:1 | 0,051598584 | 0,093070004 | 0,102770572 | 0,25326014 | 0,15549993 | 0,20751839 |
| LPC 18:0 | 0,043609941 | 0,136915258 | 0,135104289 | 0,31680721 | 0,32356204 | 0,45312513 |
| LPC 18:1 | 0,159718518 | 0,239268565 | 0,256080098 | 0,9511159 | 0,61334277 | 0,86756692 |
| LPC 18:3 | 0 | 0 | 0 | 0 | 0 | 0 |
| LPC 20:1 | 0,010696874 | 0,017728057 | 0,019606775 | 0,08474577 | 0,0510821 | 0,06654613 |
| LPC 20:3 | 0 | 0,00838563 | 0,003099039 | 0,009497 | 0,01385734 | 0,03602846 |
| LPC 20:4 | 0,002546356 | 0,021570149 | 0,011023865 | 0,08995223 | 0,04551451 | 0,07825719 |
| LPC 22:5 | 0 | 0 | 0 | 0 | 0 | 0,00548114 |
| LPC 22:6 | 0 | 0 | 0 | 0,00129893 | 0 | 0,01039109 |
| LPC O-16:0 | 0 | 0 | 0 | 0,01399116 | 0,00528687 | 0,00458382 |
| LPC O-16:1 | 0,001534031 | 0,002098182 | 0,004776469 | 0,03770738 | 0,03485328 | 0,02840719 |
| LPC O-18:0 | 0 | 0 | 0 | 0,00530633 | 0,0026511 | 0,00220106 |
| LPC O-18:1 | 0,003096189 | 0,005176551 | 0,010674334 | 0,01861131 | 0,01640105 | 0,01136926 |
| LPE 16:0 | 0 | 0,092244794 | 0,041142049 | 0 | 0 | 0 |
| LPE 16:1 | 0,023275234 | 0,023998382 | 0,017515498 | 0,03651801 | 0,04694672 | 0,03866456 |
| LPE 18:0 | 0,110518234 | 0,199160319 | 0,18240873 | 0,36849268 | 0,29102732 | 0,35834637 |
| LPE 18:1 | 0,157570748 | 0,23098968 | 0,181017053 | 0,50055858 | 0,4701873 | 0,4906955 |
| LPE 18:2 | 0,005097521 | 0 | 0 | 0,0025239 | 0,0035148 | 0,00691091 |
| LPE 20:0 | 0 | 0 | 0 | 0 | 0 | 0 |
| LPE 20:1 | 0 | 0 | 0 | 0 | 0 | 0 |
| LPE 20:3 | 0 | 0 | 0 | 0 | 0 | 0 |
| LPE 20:4 | 0,033743339 | 0,034564522 | 0,014132414 | 0,05155338 | 0,0587624 | 0,03008211 |
| LPE 22:6 | 0,01545509 | 0,002865844 | 0,003750092 | 0,01501144 | 0,01816661 | 0,01323365 |
| LPE O-14:0 | 0 | 0 | 0 | 0 | 0 | 0 |
| LPE O-16:1 | 0 | 0 | 0 | 0,01669743 | 0,00905512 | 0,01389181 |
| LPE O-18:1 | 0,020190761 | 0,029179352 | 0,024356835 | 0,02816336 | 0,04979546 | 0,02744391 |

| | | | | | | |
|---|---|---|---|---|---|---|
| LPE O-18:2 | 0 | 0 | 0 | 0,00429946 | 0,00193601 | 0 |
| LPE O-20:1 | 0 | 0 | 0 | 0 | 0 | 0 |
| LPG 14:0 | 0,004545829 | 0 | 0,004305253 | 0,00822927 | 0,01843242 | 0,03034858 |
| LPG 16:0 | 0,001601102 | 0,007072729 | 0,010555706 | 0,01450294 | 0,01436982 | 0,0260451 |
| LPG 16:1 | 0 | 0 | 0 | 0,00435458 | 0,00325163 | 0,00227958 |
| LPG 18:0 | 0 | 0 | 0 | 0,00741172 | 0,00693762 | 0,02400219 |
| LPG 18:1 | 0,012232577 | 0,011918762 | 0,018662231 | 0,24509782 | 0,14146217 | 0,20563784 |
| LPG 18:2 | 0 | 0 | 0 | 0,00189992 | 0,00163449 | 0 |
| LPG 22:6 | 0,001157077 | 0 | 0 | 0,16886007 | 0,08495397 | 0,13467578 |
| LPI 16:0 | 0,010922149 | 0,001790796 | 0,00675775 | 0 | 0,02970505 | 0,03702879 |
| LPI 16:1 | 0,011632501 | 0,002100961 | 0,002881866 | 0,04514048 | 0,06324549 | 0,03187282 |
| LPI 18:0 | 0,117622029 | 0,118685846 | 0,212567702 | 0,71249405 | 0,43847908 | 0,56762387 |
| LPI 18:1 | 0,104414124 | 0,078357628 | 0,077577074 | 0,65353565 | 0,45613448 | 0,48158296 |
| LPS 18:0 | 0,028041972 | 0,041700851 | 0,036712184 | 0,21915765 | 0,15514208 | 0,16659832 |
| LPS 18:1 | 0,027568828 | 0,023081959 | 0,017574243 | 0,34843732 | 0,25848141 | 0,26075759 |
| PA 30:1 | 0,001092699 | 0,006440417 | 0,002757691 | 0 | 0,00255919 | 0,01648701 |
| PA 32:1 | 0,024710374 | 0,064203273 | 0,050416708 | 0,01777182 | 0,01842144 | 0,01073055 |
| PA 32:2 | 0,002028398 | 0,005369177 | 0,004544462 | 0 | 0 | 0 |
| PA 34:1 | 0,030465447 | 0,080975089 | 0,056089106 | 0,00433419 | 0,01982815 | 0 |
| PA 34:2 | 0,028498179 | 0,066436733 | 0,048352442 | 0,01480771 | 0,00806622 | 0,00798652 |
| PA 36:2 | 0,034821004 | 0,065057447 | 0,049793712 | 0,00943149 | 0,01139495 | 0 |
| PA 36:3 | 0,010351377 | 0,007903982 | 0,006654904 | 0 | 0 | 0 |
| PA 36:4 | 0,003165108 | 0,009639616 | 0,007132152 | 0,34588229 | 0,04150452 | 0,05590318 |
| PA 38:3 | 0,003678411 | 0,001880775 | 0 | 0 | 0 | 0 |
| PA O-38:5 | 0,006342452 | 0,01511377 | 0,007063826 | 0,22538023 | 0,08968079 | 0,0624101 |
| PC 28:0 | 0,175394762 | 0,251035388 | 0,25862146 | 0,18519933 | 0,15987883 | 0,19210331 |
| PC 30:0 | 0,780404848 | 0,766344815 | 0,771236857 | 0,51467476 | 0,63695881 | 0,51663688 |
| PC 30:1 | 1,021117708 | 1,14664003 | 1,22353182 | 0,76657203 | 0,56423032 | 0,71253446 |
| PC 32:0 | 0,538663054 | 0,422988623 | 0,405507095 | 0,26138025 | 0,63335673 | 0,44730008 |
| PC 32:1 | 7,382739567 | 7,394606708 | 7,673070176 | 3,88141636 | 3,17536681 | 3,61085892 |
| PC 32:2 | 1,359271556 | 1,447892873 | 1,541552553 | 0,75759331 | 0,59725972 | 0,74399704 |
| PC 34:1 | 12,19441583 | 10,38357221 | 10,43412171 | 3,33024083 | 4,38736479 | 3,55526714 |

| | | | | | | |
|---|---|---|---|---|---|---|
| PC 34:2 | 6,934227653 | 6,474212419 | 6,896202316 | 2,69294161 | 2,20533518 | 2,73325288 |
| PC 34:3 | 0,361122444 | 0,354627525 | 0,388665191 | 0,15770762 | 0,14264094 | 0,17433241 |
| PC 34:4 | 0,319083305 | 0,424185771 | 0,338245328 | 0,19921865 | 0,14542353 | 0,15437933 |
| PC 36:1 | 0,432369165 | 0,086006553 | 0,025499381 | 0,08263769 | 0,56890503 | 0,35080341 |
| PC 36:2 | 10,08940211 | 8,57705507 | 8,731386718 | 2,07192256 | 2,35083102 | 2,24585333 |
| PC 36:3 | 1,159518615 | 0,963073363 | 1,06341049 | 0,32952093 | 0,31746844 | 0,37917275 |
| PC 36:4 | 1,054044201 | 1,050219589 | 0,98010326 | 0,39768352 | 0,38296695 | 0,40135092 |
| PC 36:5 | 0,498973348 | 0,551218118 | 0,532611611 | 0,19609728 | 0,16314844 | 0,1817805 |
| PC 38:1 | 0 | 0 | 0 | 0 | 0,02351545 | 0,00344381 |
| PC 38:2 | 1,13919839 | 0,944491561 | 0,903809374 | 0,11961304 | 0,2347955 | 0,14300954 |
| PC 38:3 | 0,219605004 | 0,195752795 | 0,199865883 | 0,01106309 | 0,09796231 | 0,07648069 |
| PC 38:4 | 0,36450602 | 0,290492414 | 0,307462199 | 0,07873828 | 0,16260314 | 0,14285607 |
| PC 38:5 | 0,986241218 | 0,890630446 | 0,883094259 | 0,27792827 | 0,30190795 | 0,364556 |
| PC 38:6 | 0,537978649 | 0,429297921 | 0,468155227 | 0 | 0 | 0 |
| PC 40:1 | 0,024438303 | 0,012704858 | 0,012074837 | 0 | 0 | 0 |
| PC 40:2 | 0,121438065 | 0,096083071 | 0,085900793 | 0 | 0,01887349 | 0 |
| PC 40:3 | 0,043958768 | 0,029249692 | 0,032964409 | 0 | 0,00219129 | 0 |
| PC 40:4 | 0,075190678 | 0,057096032 | 0,056790055 | 0 | 0,00789437 | 0,0105234 |
| PC 40:5 | 0,250412946 | 0,220462892 | 0,205836469 | 0,01850401 | 0,07877137 | 0,05388322 |
| PC 40:6 | 0,319326176 | 0,248153071 | 0,267567823 | 0,01844015 | 0,11021575 | 0,10918276 |
| PC 40:7 | 0,397166642 | 0,264775988 | 0,308435071 | 0,04630634 | 0,05808731 | 0,06997719 |
| PC 40:8 | 0,057229376 | 0,068079997 | 0,056401103 | 0,04332019 | 0,04602856 | 0,12237516 |
| PC 42:1 | 0 | 0,01105927 | 0,008992006 | 0 | 0 | 0 |
| PC 42:2 | 0,104148026 | 0,085481366 | 0,07340116 | 0 | 0,01910543 | 0 |
| PC 42:3 | 0,021625533 | 0,015316362 | 0,010635062 | 0 | 0 | 0 |
| PC 42:4 | 0,010602828 | 0 | 0 | 0 | 0 | 0,04246107 |
| PC 42:5 | 0,022761306 | 0,019492657 | 0,012075255 | 0 | 0 | 0 |
| PC 42:6 | 0,288329834 | 0,214232704 | 0,22684164 | 0,00271016 | 0,02337739 | 0,0128721 |
| PC 42:7 | 0,058606303 | 0,03608859 | 0,044081985 | 0 | 0 | 0 |
| PC 42:8 | 0,00353135 | 0,002904652 | 0 | 0 | 0 | 0 |
| PC 42:9 | 0,073241732 | 0,06631209 | 0,068573262 | 0,00269371 | 0,02816937 | 0,0157969 |
| PC 44:2 | 0,074416306 | 0,064593841 | 0,060698935 | 0 | 0,00950677 | 0 |

| | | | | | | |
|---|---|---|---|---|---|---|
| PC 44:3 | 0,022717215 | 0,010334508 | 0,011521235 | 0 | 0 | 0 |
| PC 44:6 | 0,042616094 | 0,021828472 | 0,021309857 | 0 | 0 | 0 |
| PC 44:7 | 0,004047998 | 0 | 0 | 0 | 0 | 0 |
| PC 44:9 | 0,084937669 | 0,069133939 | 0,070297904 | 0 | 0,02671529 | 0,00238822 |
| PC O-30:0 | 0 | 0 | 0 | 0 | 0,00885113 | 0,00350697 |
| PC O-30:1 | 0,007025572 | 0,006054433 | 0,007784743 | 0,0022738 | 0,01348106 | 0,00672417 |
| PC O-32:0 | 0 | 0 | 0 | 0,00478021 | 0,07939675 | 0,063948 |
| PC O-32:1 | 0,015478612 | 0,017891131 | 0,018741558 | 0,03097434 | 0,11259107 | 0,09661178 |
| PC O-32:2 | 0,031864989 | 0,030508894 | 0,032125213 | 0,00578133 | 0,02806714 | 0,01306912 |
| PC O-34:0 | 0 | 0 | 0 | 0 | 0,00659062 | 0,00277839 |
| PC O-34:1 | 0,004544384 | 0,020179262 | 0,01811839 | 0,10755767 | 0,22709391 | 0,16963091 |
| PC O-34:2 | 0,005158675 | 0,016168134 | 0,012438452 | 0,04205038 | 0,13091704 | 0,1025844 |
| PC O-36:1 | 0 | 0 | 0 | 0 | 0,01342005 | 0 |
| PC O-36:2 | 0,046770915 | 0,041331931 | 0,039635392 | 0,00208947 | 0,04284722 | 0,02007072 |
| PC O-36:3 | 0,009777753 | 0,007663907 | 0,007317428 | 0 | 0,01550647 | 0,00388467 |
| PC O-36:4 | 0,005708551 | 0 | 0,003094107 | 0,00339681 | 0,01906065 | 0,01941443 |
| PC O-36:5 | 0,010162478 | 0,002408877 | 0,001696983 | 0 | 0,01001647 | 0,00636686 |
| PC O-38:1 | 0 | 0 | 0,001782503 | 0 | 0,01357753 | 0 |
| PC O-38:2 | 0 | 0 | 0 | 0 | 0 | 0 |
| PC O-38:4 | 0,001259687 | 0 | 0 | 0 | 0,01096741 | 0,00252498 |
| PC O-38:6 | 0 | 0 | 0 | 0 | 0 | 0 |
| PC O-40:1 | 0,005361028 | 0,004869924 | 0,006450976 | 0 | 0,01857294 | 0,00493107 |
| PC O-40:5 | 0 | 0 | 0 | 0 | 0,003749 | 0 |
| PC O-40:6 | 0 | 0,002591704 | 0,003025083 | 0,00103934 | 0,02196439 | 0,01256179 |
| PC O-40:7 | 0 | 0 | 0 | 0 | 0,01128359 | 0,00428296 |
| PC O-42:5 | 0 | 0 | 0 | 0 | 0,01434505 | 0,03095832 |
| PC O-42:6 | 0 | 0 | 0 | 0 | 0 | 0 |
| PC O-44:4 | 0,036638388 | 0,043614783 | 0,045918532 | 0 | 0,0069943 | 0 |
| PC O-44:5 | 0 | 0 | 0 | 0 | 0,00323086 | 0,0114865 |
| PE 32:1 | 0,25777014 | 0,291080864 | 0,269984694 | 0,14050455 | 0,19036391 | 0,07191524 |
| PE 34:1 | 1,158588356 | 0,974608811 | 0,909819434 | 0,36446126 | 0,66758668 | 0,29622597 |
| PE 34:2 | 0,890515427 | 0,900626003 | 0,920639861 | 0,59938398 | 0,52801975 | 0,43577569 |

| | | | | | | |
|---|---|---|---|---|---|---|
| PE 34:3 | 0,070715356 | 0,026330811 | 0,046824205 | 0,0032604 | 0,00407821 | 0 |
| PE 36:1 | 0,950773912 | 0,628721004 | 0,569175746 | 0,22633153 | 0,41321901 | 0,16093144 |
| PE 36:2 | 2,97007063 | 2,309997451 | 2,325301972 | 1,01357402 | 1,41030889 | 0,69562492 |
| PE 36:3 | 0,28485263 | 0,23937926 | 0,252118064 | 0,11001098 | 0,12097965 | 0,06629121 |
| PE 36:4 | 0,2746657 | 0,256121769 | 0,275182804 | 0,13698274 | 0,14677033 | 0,0650002 |
| PE 36:5 | 0,09708362 | 0,104027287 | 0,106254488 | 0,03484212 | 0,03061898 | 0,01634282 |
| PE 38:2 | 0,212133659 | 0,146726546 | 0,152540562 | 0,00911481 | 0,08910885 | 0,00233425 |
| PE 38:3 | 0,134933228 | 0,075168185 | 0,074340377 | 0 | 0,01894702 | 0 |
| PE 38:4 | 0,954138715 | 0,674564004 | 0,684429645 | 0,15251851 | 0,31785004 | 0,1102349 |
| PE 38:5 | 0,846189805 | 0,674018485 | 0,714675681 | 0,29464885 | 0,29471248 | 0,21404912 |
| PE 38:6 | 0,250623523 | 0,178567222 | 0,210752837 | 0,08400412 | 0,03262167 | 0,0244897 |
| PE 40:2 | 0,019361674 | 0,002128173 | 0 | 0 | 0,00204569 | 0 |
| PE 40:5 | 0,122139533 | 0,067585767 | 0,076128277 | 0 | 0,02657679 | 0 |
| PE 40:6 | 0,485532372 | 0,249867079 | 0,279211292 | 0,02010699 | 0,01540971 | 0 |
| PE 40:7 | 0,06692262 | 0,097376407 | 0,125001437 | 0,02094363 | 0 | 0,0040106 |
| PG 32:1 | 0,001839271 | 0,035093532 | 0,024941819 | 0,13621768 | 0,08908491 | 0,10483875 |
| PG 32:2 | 0 | 0 | 0 | 0,056545 | 0,03340091 | 0,03679139 |
| PG 34:1 | 0,098618917 | 0,181679681 | 0,138026121 | 0,34524801 | 0,292509 | 0,21748226 |
| PG 34:2 | 0,025884171 | 0,0460456 | 0,04600133 | 0,91394241 | 0,51179931 | 0,59984685 |
| PG 34:3 | 0 | 0 | 0 | 0,08524098 | 0,05325753 | 0,05773473 |
| PG 36:2 | 0,073585735 | 0,092375309 | 0,084646995 | 2,95817172 | 1,58314856 | 1,59798755 |
| PG 36:3 | 0,007368221 | 0,007303247 | 0,009754031 | 0,53062296 | 0,27592232 | 0,31812312 |
| PG 36:4 | 0 | 0 | 0 | 0,14278612 | 0,08842857 | 0,10781506 |
| PG 36:5 | 0 | 0 | 0 | 0,02933703 | 0,02506156 | 0,02594902 |
| PG 38:3 | 0,00104889 | 0 | 0 | 0,1854822 | 0,10688695 | 0,09452646 |
| PG 38:4 | 0,002807645 | 0,002346164 | 0,003458099 | 0,39505512 | 0,25920164 | 0,27362414 |
| PG 38:5 | 0 | 0,001884993 | 0,001694428 | 0,29521042 | 0,16975544 | 0,18449153 |
| PG 38:6 | 0,001192288 | 0,00324206 | 0,004744383 | 0,3832336 | 0,217797 | 0,25105719 |
| PG 38:7 | 0,001876645 | 0,001880514 | 0,002620916 | 0,42551052 | 0,25350409 | 0,27910306 |
| PG 40:5 | 0 | 0 | 0 | 0,12978652 | 0,0762668 | 0,07504114 |
| PG 40:6 | 0 | 0 | 0 | 0,37911802 | 0,22839626 | 0,21521688 |
| PG 40:7 | 0,069957744 | 0,060004964 | 0,067460122 | 3,50336361 | 1,90378068 | 1,97521095 |

| | | | | | | |
|---|---|---|---|---|---|---|
| PG 40:8 | 0,001316779 | 0 | 0 | 0,28779866 | 0,16309363 | 0,1807031 |
| PG 42:10 | 0 | 0 | 0 | 0,17580011 | 0,1124049 | 0,10824023 |
| PG 42:7 | 0 | 0 | 0 | 0,06217368 | 0,03311353 | 0,01757059 |
| PG 42:8 | 0 | 0 | 0 | 0,14054284 | 0,08575724 | 0,07943802 |
| PG 42:9 | 0 | 0 | 0 | 0,27463022 | 0,18280349 | 0,18440254 |
| PG 44:10 | 0 | 0 | 0 | 0,09613006 | 0,05732023 | 0,04126224 |
| PG 44:11 | 0,001558293 | 0 | 0 | 0,2808581 | 0,15470134 | 0,13807637 |
| PG 44:12 | 0,032137177 | 0,019999717 | 0,026267635 | 1,5047537 | 0,83296981 | 0,82439207 |
| PI 30:0 | 0,001888546 | 0,020858201 | 0,008491604 | 0,00125807 | 0,00456881 | 0 |
| PI 30:1 | 0 | 0,009546393 | 0,004237516 | 0 | 0 | 0 |
| PI 32:0 | 0,042633774 | 0,147286011 | 0,09619895 | 0,06566358 | 0,14543465 | 0,03964737 |
| PI 32:1 | 0,137053969 | 0,350223974 | 0,252171089 | 0,30414367 | 0,20558941 | 0,17172148 |
| PI 34:1 | 0,768112545 | 1,346030007 | 0,978549247 | 1,19907957 | 1,08953612 | 0,49794679 |
| PI 34:2 | 0,245587381 | 0,567483654 | 0,454286585 | 0,49701792 | 0,3143052 | 0,28282558 |
| PI 36:1 | 0,779153685 | 0,873628671 | 0,620877251 | 0,92857831 | 0,97439071 | 0,33744081 |
| PI 36:2 | 0,601514779 | 1,015463793 | 0,765126288 | 0,77114367 | 0,5829699 | 0,3128771 |
| PI 36:3 | 0,072623501 | 0,140874741 | 0,121675218 | 0,67793593 | 0,292532 | 0,26985442 |
| PI 36:4 | 0,055935492 | 0,115707765 | 0,073743491 | 0,11195436 | 0,1026941 | 0,04461526 |
| PI 36:5 | 0,004651814 | 0,027147791 | 0,009714354 | 0 | 0,008727 | 0 |
| PI 38:1 | 0,041304932 | 0,050054647 | 0,026932737 | 0,01615272 | 0,03869601 | 0,00281812 |
| PI 38:2 | 0,087512497 | 0,099071944 | 0,07229958 | 0,03232418 | 0,08780136 | 0,00366067 |
| PI 38:3 | 0,136846703 | 0,157360832 | 0,118436118 | 0,11015273 | 0,10635438 | 0,01608509 |
| PI 38:4 | 0,164666861 | 0,199863551 | 0,143490914 | 0,20432415 | 0,14839581 | 0,05362582 |
| PI 38:5 | 0,099976062 | 0,177094179 | 0,125009199 | 0,13351409 | 0,09898695 | 0,04964846 |
| PI 38:6 | 0,098951316 | 0,185373403 | 0,146265071 | 0,14306623 | 0,099709 | 0,06978073 |
| PI 40:3 | 0,082902886 | 0,253772162 | 0,161351029 | 2,60278965 | 1,37355183 | 0,74772131 |
| PI 40:4 | 0,025927848 | 0,037566191 | 0,024900113 | 0,27126083 | 0,09320332 | 0,04730581 |
| PI 40:5 | 0,046189277 | 0,070917533 | 0,049146334 | 0,00770484 | 0,01543953 | 0 |
| PI 40:6 | 0,138254625 | 0,177710985 | 0,13545447 | 0,09029297 | 0,09816694 | 0,01910386 |
| PI 40:7 | 0,054005702 | 0,108991566 | 0,087717147 | 0,01733375 | 0,03396197 | 0,01293765 |
| PI 42:5 | 0,005618781 | 0,001861158 | 0,002625312 | 0 | 0 | 0 |
| PI 42:6 | 0 | 0 | 0 | 0 | 0,00213173 | 0 |

| | | | | | | |
|---|---|---|---|---|---|---|
| PS 32:1 | 0,031589308 | 0,105336874 | 0,076247934 | 0,08233541 | 0,11467525 | 0,09308056 |
| PS 34:1 | 0,201888339 | 0,456060854 | 0,320682123 | 0,29134742 | 0,50762388 | 0,25303006 |
| PS 34:2 | 0,04748535 | 0,114941222 | 0,09029984 | 0,12626384 | 0,13028293 | 0,11254518 |
| PS 36:1 | 0,313810285 | 0,478483384 | 0,33193172 | 0,40050804 | 0,65002415 | 0,258544 |
| PS 36:2 | 0,176582984 | 0,378386167 | 0,272087414 | 0,29385704 | 0,36770209 | 0,19638782 |
| PS 36:4 | 0,016366957 | 0,062567664 | 0,030877646 | 0,00504421 | 0,04944683 | 0,00979329 |
| PS 38:2 | 0,023519195 | 0,043269611 | 0,028138226 | 0,00777097 | 0,03882766 | 0,00329106 |
| PS 38:4 | 0,039956179 | 0,082364976 | 0,04771375 | 0,04880257 | 0,08682818 | 0,03543608 |
| PS 40:2 | 0,004079062 | 0,004674638 | 0,003396536 | 0 | 0,0047052 | 0 |
| PS 40:6 | 0 | 0 | 0 | 0 | 0 | 0 |
| PS 40:7 | 0,006521216 | 0,030408168 | 0,018494155 | 0,00650104 | 0,01874318 | 0,01105494 |
| PS 42:7 | 0 | 0,006530931 | 0,0033823 | 0,01777009 | 0,00546583 | 0,00408479 |
| SM 32:0;2 | 0 | 0 | 0 | 0 | 0 | 0 |
| SM 32:1;2 | 0,108581064 | 0,109905197 | 0,118555354 | 0,27900123 | 0,20106267 | 0,26255318 |
| SM 32:2;2 | 0 | 0,001729379 | 0 | 0,00359791 | 0,00250507 | 0,01324274 |
| SM 34:0;2 | 0,20086563 | 0,173834629 | 0,169444254 | 0,1494815 | 0,27846194 | 0,20173911 |
| SM 34:1;2 | 1,920194552 | 1,905554934 | 1,874826507 | 2,22743394 | 3,31473811 | 2,59940513 |
| SM 34:2;2 | 0,141323773 | 0,157509263 | 0,162878684 | 0,45807057 | 0,32179189 | 0,47616241 |
| SM 36:0;2 | 0 | 0 | 0 | 0 | 0,00714447 | 0,00230336 |
| SM 36:1;2 | 0,082224185 | 0,097587214 | 0,099448988 | 0,0900563 | 0,31420509 | 0,26388122 |
| SM 36:2;2 | 0,011700645 | 0,021175564 | 0,020604415 | 0,02546036 | 0,08233877 | 0,10569422 |
| SM 38:1;2 | 0,008670412 | 0,028107515 | 0,018312902 | 0,01386865 | 0,14373661 | 0,11806018 |
| SM 38:2;2 | 0 | 0 | 0 | 0 | 0,01576807 | 0,00432099 |
| SM 40:0;2 | 0 | 0 | 0 | 0 | 0,00566943 | 0,00364476 |
| SM 40:1;2 | 0,146052143 | 0,1665279 | 0,151484457 | 0,15111455 | 0,41833588 | 0,36823365 |
| SM 40:2;2 | 0,071742176 | 0,090453771 | 0,085489898 | 0,04492957 | 0,23420866 | 0,15466412 |
| SM 42:1;2 | 0,06112491 | 0,089778879 | 0,098560034 | 0,07916603 | 0,11623437 | 0,1956529 |
| SM 42:2;2 | 0,904357887 | 0,826164767 | 0,734577144 | 0,562308 | 1,45267045 | 0,94349179 |
| SM 42:3;2 | 0,102505285 | 0,113845082 | 0,096745891 | 0,04066767 | 0,16810697 | 0,11906286 |
| SM 44:2;2 | 0,128936462 | 0,102318437 | 0,094108829 | 0,02436969 | 0,11859845 | 0,08280659 |

| Experiment no. | 3 | 3 | 3 | 3 | 3 | 3 |
|---|---|---|---|---|---|---|
| Sample type | whole cell lysate | whole cell lysate | whole cell lysate | lysosomes | lysosomes | lysosomes |
| Treatment | control | GalSph | GlcSph | control | GalSph | GlcSph |
| CE 14:0 | 0,00062914 | 0,07020892 | 0,01641465 | 0,01300302 | 0,26774101 | 0,31594507 |
| CE 16:0 | 0,03738913 | 0,70516566 | 0,43275984 | 0,63573566 | 2,24870694 | 2,55166288 |
| CE 16:1 | 0,03178367 | 0,47664999 | 0,27998328 | 0,71323635 | 1,74645777 | 1,85133709 |
| CE 18:0 | 0 | 0,03414785 | 0,01095576 | 0,03488649 | 0,29436715 | 0,36056726 |
| CE 18:1 | 0,20206983 | 1,44164738 | 0,96233576 | 1,66808759 | 4,87623377 | 5,57713995 |
| CE 18:2 | 0,01090859 | 0,41937886 | 0,23357241 | 0,36595837 | 1,19642052 | 1,29568454 |
| CE 20:3 | 0 | 0,00686787 | 0,0021305 | 0,0023934 | 0,07266725 | 0,07158403 |
| CE 20:4 | 0,00196163 | 0,36959568 | 0,17508387 | 0,23334925 | 0,89503625 | 0,80040866 |
| CE 20:5 | 0 | 0,07199653 | 0,00572697 | 0,00838431 | 0,24183385 | 0,21040638 |
| CE 22:6 | 0 | 0,11706956 | 0,01532092 | 0 | 0,53305271 | 0,61714468 |
| Cer 32:1;2 | 0,00574533 | 0,01916009 | 0,00598086 | 0 | 0,00433948 | 0,00081066 |
| Cer 34:1;2 | 0,14082989 | 0,26978417 | 0,20023239 | 0,36466728 | 0,38138988 | 0,29095974 |
| Cer 34:2;2 | 0,02151905 | 0,00368003 | 0,00168767 | 0,0069548 | 0,00542155 | 0,01015371 |
| Cer 36:1;2 | 0,00413521 | 0,02626998 | 0,01228981 | 0,02974242 | 0,06475549 | 0,0644749 |
| Cer 36:2;2 | 0,00018709 | 0,0011705 | 0 | 0,00115487 | 0,00188875 | 0,00660843 |
| Cer 40:1;2 | 0,01047627 | 0,06755225 | 0,03255517 | 0,02144902 | 0,05285534 | 0,05134532 |
| Cer 40:2;2 | 0,00588718 | 0,02320566 | 0,00891508 | 0,00648443 | 0,01646994 | 0,0235612 |
| Cer 42:1;2 | 0,07715761 | 0,20206335 | 0,15069009 | 0,1080919 | 0,12579192 | 0,10792463 |
| Cer 42:2;2 | 0,19269885 | 0,46200095 | 0,36901296 | 0,25123035 | 0,30314369 | 0,27019081 |
| Cer 42:3;2 | 0,04995053 | 0,09640651 | 0,0817307 | 0,0153245 | 0,02180122 | 0,03194155 |
| Cer 44:1;2 | 0,01267261 | 0,0005564 | 0,0001623 | 0,00035598 | 0 | 0,00027739 |
| Cer 44:2;2 | 0,048007 | 0,0620166 | 0,02852811 | 0,0071029 | 0,01091118 | 0,00434229 |
| Chol : | 22,9758432 | 23,3425425 | 23,7100985 | 44,2991644 | 39,9643666 | 34,902896 |
| CL 62:2 | 0,07394243 | 1,34512639 | 0,90746377 | 0 | 0 | 0 |
| CL 64:4 | 0,0416368 | 0,05487721 | 0,08625458 | 0 | 0 | 0 |
| CL 66:2 | 0,01646016 | 0 | 0 | 0 | 0 | 0 |
| CL 66:4 | 0 | 0 | 0 | 0 | 0 | 0 |
| CL 66:5 | 0,05225706 | 0,01923356 | 0,06210799 | 0 | 0 | 0 |
| CL 68:2 | 0 | 0 | 0 | 0 | 0 | 0 |

| | | | | | | |
|---|---|---|---|---|---|---|
| CL 68:3 | 0 | 0 | 0,04390274 | 0 | 0 | 0 |
| CL 68:4 | 1,79603786 | 3,34890081 | 2,31662553 | 0 | 0 | 0 |
| CL 68:5 | 0 | 0,71216467 | 0 | 0 | 0 | 0 |
| CL 70:4 | 1,12733116 | 1,97908736 | 1,40647213 | 0 | 0 | 0 |
| CL 70:5 | 0 | 0 | 0 | 0 | 0 | 0 |
| CL 70:6 | 0,01858298 | 0 | 0,05656814 | 0 | 0 | 0 |
| CL 72:4 | 0 | 0 | 0 | 0 | 0 | 0 |
| CL 72:5 | 0,03831905 | 0,16391857 | 0,15006507 | 0 | 0 | 0 |
| CL 72:6 | 0,00814548 | 0 | 0,04534582 | 0 | 0 | 0 |
| DAG 28:0 | 0 | 0 | 0 | 0 | 0 | 0 |
| DAG 30:0 | 0 | 0 | 0 | 0 | 0 | 0 |
| DAG 30:1 | 0,01210444 | 0 | 0 | 0 | 0 | 0 |
| DAG 32:0 | 0 | 0 | 0 | 0 | 0 | 0 |
| DAG 32:1 | 0,12359618 | 0,08867836 | 0,10222183 | 0 | 0 | 0 |
| DAG 32:2 | 0,03617526 | 0,0319638 | 0,02878993 | 0 | 0 | 0 |
| DAG 34:1 | 0,22534645 | 0,12736932 | 0,16420403 | 0 | 0 | 0 |
| DAG 34:2 | 0,21692412 | 0,18923843 | 0,20089171 | 0,00589222 | 0,00731963 | 0,02674712 |
| DAG 34:3 | 0,01583608 | 0,00382192 | 0,00432547 | 0 | 0 | 0 |
| DAG 36:1 | 0 | 0 | 0 | 0 | 0 | 0 |
| DAG 36:2 | 0,30507689 | 0,28853049 | 0,29356284 | 0 | 0 | 0,0171841 |
| DAG 36:3 | 0,08900172 | 0,04778677 | 0,05219975 | 0,00058862 | 0,00293566 | 0,00249385 |
| DAG 36:4 | 0,0606886 | 0,02162994 | 0,02189967 | 0,01407353 | 0,00059682 | 0,00450633 |
| DAG 38:2 | 0,02286262 | 0 | 0 | 0 | 0 | 0 |
| DAG 38:3 | 0,02296452 | 0,01914113 | 0,01381916 | 0 | 0,00043814 | 0,00094498 |
| DAG 38:4 | 0 | 0 | 0 | 0 | 0 | 0 |
| DAG 38:5 | 0 | 0 | 0 | 0 | 0 | 0 |
| DAG 38:6 | 0 | 0 | 0 | 0 | 0 | 0 |
| DAG 40:5 | 0 | 0 | 0 | 0 | 0 | 0 |
| DAG 40:6 | 0 | 0 | 0 | 0 | 0 | 0 |
| DAG 42:2 | 0,00617101 | 0,01383382 | 0,00325933 | 0 | 0 | 0 |
| diHexCer 42:2;2 | 0 | 0 | 0 | 0 | 0 | 0 |
| HexCer 32:1;2 | 0 | 0 | 0 | 0 | 0 | 0 |

| | | | | | | |
|---|---|---|---|---|---|---|
| HexCer 34:0;2 | 0 | 0 | 0 | 0 | 0 | 0 |
| HexCer 34:1;2 | 0,13809175 | 0,11145849 | 0,12300112 | 0,43121536 | 0,63882652 | 0,49627892 |
| HexCer 34:2;2 | 0 | 0,00096912 | 0 | 0 | 0,00569144 | 0,00655967 |
| HexCer 36:1;2 | 0 | 0 | 0 | 0 | 0 | 0 |
| HexCer 36:2;2 | 0 | 0,00954745 | 0,00128207 | 0 | 0,00991501 | 0,00794776 |
| HexCer 40:1;2 | 0 | 0,02854862 | 0 | 0 | 0,08474301 | 0,12254545 |
| HexCer 40:2;2 | 0 | 0,00120581 | 0 | 0 | 0,02275737 | 0,02592616 |
| HexCer 42:1;2 | 0,12162949 | 0,18787733 | 0,10927502 | 0,28790562 | 0,36302478 | 0,3466976 |
| HexCer 42:2;2 | 0,38440785 | 0,45549838 | 0,3546411 | 1,0815648 | 1,28344504 | 1,18497179 |
| HexCer 42:3;2 | 0,00182675 | 0,01296026 | 0,01175987 | 0,02651112 | 0,0698865 | 0,08522134 |
| HexCer 44:1;2 | 0,00767026 | 0,04033107 | 0,00412236 | 0,0864677 | 0,10323984 | 0,09809461 |
| HexCer 44:2;2 | 0,12498071 | 0,13193002 | 0,08143775 | 0,24602244 | 0,30795364 | 0,29326045 |
| LPA 14:0 | 0,00550784 | 0,04501823 | 0,01709781 | 0 | 0,09537519 | 0,07033286 |
| LPA 16:0 | 0,00876242 | 0,03226954 | 0,02450665 | 0,01716605 | 0,01833295 | 0,02504864 |
| LPA 16:1 | 0,00098636 | 0,00802313 | 0,00745683 | 0,00157286 | 0,00231883 | 0,00431006 |
| LPA 18:0 | 0,01260282 | 0,02573158 | 0,0313043 | 0,00671786 | 0,01359806 | 0,01047271 |
| LPA 18:1 | 0,03176521 | 0,11236022 | 0,08571593 | 0,06622564 | 0,09236885 | 0,11978406 |
| LPA 18:2 | 0,00109193 | 0,00260926 | 0,00280893 | 0 | 0,00061297 | 0,00092958 |
| LPC 14:0 | 0,01296803 | 0,07806206 | 0,04974692 | 0,06672781 | 0,04659592 | 0,05877221 |
| LPC 16:0 | 0,11276378 | 0,50061573 | 0,34457701 | 1,27542328 | 0,90287958 | 0,86968671 |
| LPC 16:1 | 0,04743378 | 0,17917134 | 0,11699898 | 0,43844629 | 0,32730996 | 0,29633166 |
| LPC 18:0 | 0,02633073 | 0,14988551 | 0,1012603 | 0,24881412 | 0,29422409 | 0,23775105 |
| LPC 18:1 | 0,13102072 | 0,48154412 | 0,31431484 | 1,59681724 | 1,21034401 | 1,13300132 |
| LPC 18:3 | 0,01243251 | 0,05518013 | 0,0183149 | 0,1725395 | 0,11041771 | 0,12220516 |
| LPC 20:1 | 0,01090884 | 0,04705169 | 0,03193616 | 0,12316275 | 0,08842209 | 0,08973925 |
| LPC 20:3 | 0,00098445 | 0,00850076 | 0,00935088 | 0,04325896 | 0,06193279 | 0,06768553 |
| LPC 20:4 | 0,01682976 | 0,05795058 | 0,04698958 | 0,27648385 | 0,21822008 | 0,18983987 |
| LPC 22:5 | 0 | 0,0003746 | 0 | 0,00196034 | 0,03931267 | 0,04701933 |
| LPC 22:6 | 0,00017649 | 0 | 0 | 0,0160764 | 0,05315992 | 0,065186 |
| LPC O-16:0 | 0 | 0 | 0 | 0,00888726 | 0,0002131 | 0 |
| LPC O-16:1 | 0,00604596 | 0,01127623 | 0,01116619 | 0,10408345 | 0,07320243 | 0,05903862 |
| LPC O-18:0 | 0,00016674 | 0 | 0 | 0,00461218 | 0,00249181 | 0,00377924 |

| | | | | | | |
|---|---|---|---|---|---|---|
| LPC O-18:1 | 0 | 0 | 0 | 0 | 0 | 0 |
| LPE 16:0 | 0,03598311 | 0,02900175 | 0,0149015 | 0,07529726 | 0 | 0,08255982 |
| LPE 16:1 | 0,00997639 | 0,03265412 | 0,02556251 | 0,11206148 | 0,10969188 | 0,09221506 |
| LPE 18:0 | 0,05693735 | 0,23949037 | 0,15803867 | 0,23836425 | 0,19952648 | 0,20482743 |
| LPE 18:1 | 0,07299294 | 0,28115238 | 0,19526802 | 0,62895378 | 0,61339526 | 0,56654146 |
| LPE 18:2 | 0,00056397 | 0,00162244 | 0,00211777 | 0,0087204 | 0,0077758 | 0,00618862 |
| LPE 20:0 | 0,00326384 | 0,00485348 | 0,00447389 | 0,0033643 | 0,00568781 | 0,00465205 |
| LPE 20:1 | 0,00326748 | 0,01830576 | 0,01505916 | 0,02233835 | 0,02222752 | 0,02475333 |
| LPE 20:3 | 0,00010516 | 0,00031304 | 0 | 0,00224669 | 0,00382866 | 0,00393822 |
| LPE 20:4 | 0,01219592 | 0,02193234 | 0,01984896 | 0,06205269 | 0,07394146 | 0,06262743 |
| LPE 22:6 | 0,00328065 | 0,00627241 | 0,00540607 | 0,03113128 | 0,03185307 | 0,03044296 |
| LPE O-14:0 | 0 | 0 | 0,00092202 | 0,00318677 | 0,01172931 | 0,00140217 |
| LPE O-16:1 | 0,00138367 | 0,00192077 | 0,00187453 | 0,04028559 | 0,01474249 | 0,01366804 |
| LPE O-18:1 | 0,04981662 | 0,06445544 | 0,06453509 | 0,05787739 | 0,12837526 | 0,05881006 |
| LPE O-18:2 | 0 | 0,00036537 | 0 | 0,0145613 | 0,00938743 | 0,00605104 |
| LPE O-20:1 | 0,00172065 | 0,00102277 | 0,00075023 | 0,07677454 | 0,03750886 | 0,04335033 |
| LPG 14:0 | 0 | 0,0003617 | 0 | 0 | 0,00339088 | 0,00367097 |
| LPG 16:0 | 0,00078848 | 0,01386309 | 0,00893149 | 0,00756722 | 0,00977492 | 0,00988034 |
| LPG 16:1 | 0,00012854 | 0,00022524 | 0 | 0,00530475 | 0,00781092 | 0,00463024 |
| LPG 18:0 | 0,00010573 | 0,00108034 | 0,00035713 | 0,00096227 | 0,00438245 | 0,00280281 |
| LPG 18:1 | 0,01343094 | 0,03537887 | 0,02320742 | 0,26062005 | 0,23412729 | 0,19491009 |
| LPG 18:2 | 0,00028603 | 0 | 0 | 0,00281555 | 0,00205896 | 0,00264504 |
| LPG 22:6 | 0,00178635 | 0 | 0,00037106 | 0,14139583 | 0,12677423 | 0,1022989 |
| LPI 16:0 | 0,00255836 | 0,00784327 | 0,01787602 | 0,02299972 | 0,04171743 | 0,08976903 |
| LPI 16:1 | 0 | 0 | 0 | 0 | 0 | 0 |
| LPI 18:0 | 0,15949051 | 0,57744425 | 0,45917388 | 0,87131955 | 1,04230941 | 1,25506514 |
| LPI 18:1 | 0,09800271 | 0,24437688 | 0,27607581 | 0,59791382 | 1,08855702 | 1,21954734 |
| LPS 18:0 | 0,01467535 | 0,05626846 | 0,04458684 | 0,188225 | 0,16457069 | 0,19291519 |
| LPS 18:1 | 0,01494557 | 0,03084858 | 0,03768683 | 0,53479631 | 0,41271557 | 0,3717843 |
| PA 30:1 | 0 | 0,02679134 | 0,0130763 | 0,08141624 | 0,30805174 | 0,14090659 |
| PA 32:1 | 0,02462719 | 0,01392351 | 0,02610294 | 0,04953545 | 0,13626266 | 0,0874528 |
| PA 32:2 | 0,00855761 | 0,00456178 | 0,00888797 | 0 | 0,00145832 | 0 |

| | | | | | | |
|---|---|---|---|---|---|---|
| PA 34:1 | 0,02603065 | 0,02231036 | 0,03375888 | 0,00870872 | 0,00061729 | 0,00609644 |
| PA 34:2 | 0,0424514 | 0,02451746 | 0,03749385 | 0,00930924 | 0 | 0,00189327 |
| PA 36:2 | 0,03718875 | 0,02430568 | 0,03673266 | 0,00581202 | 0 | 0,00244342 |
| PA 36:3 | 0,03186374 | 0,00148123 | 0,0094723 | 0 | 0 | 0 |
| PA 36:4 | 0,01780153 | 0 | 0 | 0 | 0 | 0,01375929 |
| PA 38:3 | 0,00131863 | 0 | 0,0006989 | 0 | 0 | 0 |
| PA O-38:5 | 0 | 0 | 0 | 0 | 0 | 0 |
| PC 28:0 | 0,13758885 | 0,15206231 | 0,15066051 | 0,18825666 | 0,13103617 | 0,1179851 |
| PC 30:0 | 0,56890273 | 0,46461539 | 0,50557973 | 0,93180438 | 0,61424632 | 0,62955553 |
| PC 30:1 | 1,11123095 | 1,12174275 | 1,05622172 | 0,78744641 | 0,5045467 | 0,48430426 |
| PC 32:0 | 0,33247464 | 0,42520964 | 0,35950537 | 0,85058767 | 0,7798746 | 0,6744722 |
| PC 32:1 | 7,19945458 | 5,21594875 | 5,5425429 | 3,99884546 | 2,54103793 | 2,68820186 |
| PC 32:2 | 2,14794672 | 2,05635937 | 1,93523121 | 0,94167789 | 0,67577947 | 0,66290576 |
| PC 34:1 | 9,64010066 | 7,46217412 | 7,55201252 | 4,49377306 | 3,32109724 | 3,52585331 |
| PC 34:2 | 10,0137965 | 6,81357316 | 7,01825421 | 3,06903188 | 2,1658894 | 2,35766301 |
| PC 34:3 | 0,52060151 | 0,39356969 | 0,40487179 | 0,20194012 | 0,15789639 | 0,15633313 |
| PC 34:4 | 0,48927544 | 0,50799498 | 0,58348203 | 0,30410954 | 0,21020261 | 0,20404655 |
| PC 36:1 | 0 | 0,32847707 | 0,04743559 | 0,39221537 | 0,61018183 | 0,5946608 |
| PC 36:2 | 11,3277802 | 8,07206011 | 8,03249467 | 2,39760705 | 1,81640331 | 2,08178733 |
| PC 36:3 | 1,40137796 | 0,85630886 | 0,89390748 | 0,31230128 | 0,25506373 | 0,302861 |
| PC 36:4 | 1,02358022 | 0,85690339 | 0,92403877 | 0,43009552 | 0,33467824 | 0,35132008 |
| PC 36:5 | 0,79972148 | 0,76152684 | 0,85860002 | 0,29103693 | 0,21044976 | 0,20999982 |
| PC 38:1 | 0 | 0 | 0 | 0,01114906 | 0,03276134 | 0 |
| PC 38:2 | 1,18869169 | 1,20221834 | 1,04118131 | 0,23391665 | 0,19605645 | 0,21517385 |
| PC 38:3 | 0,32519338 | 0,27488922 | 0,25430192 | 0,06065413 | 0,12375372 | 0,14576436 |
| PC 38:4 | 0,33738605 | 0,23481281 | 0,19782725 | 0,1056505 | 0,15828952 | 0,17112844 |
| PC 38:5 | 1,37437858 | 1,05255883 | 1,16447197 | 0,31518441 | 0,27408819 | 0,34148753 |
| PC 38:6 | 0,60225926 | 0,4300812 | 0,42723417 | 0 | 0 | 0 |
| PC 40:1 | 0,04378637 | 0,06291677 | 0,04291508 | 0,00420689 | 0,01414237 | 0,01622726 |
| PC 40:2 | 0,16854701 | 0,20439219 | 0,16499225 | 0,01722883 | 0,02482629 | 0,03542502 |
| PC 40:3 | 0,05618764 | 0,05642408 | 0,04770663 | 0 | 0,00039881 | 0,00288231 |
| PC 40:4 | 0,10897945 | 0,09283917 | 0,08063714 | 0,00273811 | 0,00309735 | 0,02506015 |

| | | | | | | |
|---|---|---|---|---|---|---|
| PC 40:5 | 0,35202494 | 0,30756746 | 0,28560847 | 0,06026603 | 0,0878633 | 0,11650531 |
| PC 40:6 | 0,41719232 | 0,26936649 | 0,26000161 | 0,02005395 | 0,07068116 | 0,11028334 |
| PC 40:7 | 0,54838101 | 0,31267359 | 0,31864636 | 0,07309975 | 0,05658203 | 0,09052275 |
| PC 40:8 | 0 | 0 | 0 | 0 | 0 | 0 |
| PC 42:1 | 0,02346383 | 0,04582153 | 0,02977499 | 0,0162152 | 0,02056993 | 0,02069541 |
| PC 42:2 | 0,19240655 | 0,26950778 | 0,19802776 | 0,06754563 | 0,06377256 | 0,06745088 |
| PC 42:3 | 0,03703566 | 0,04647818 | 0,03925588 | 0 | 0,00047103 | 0,00039374 |
| PC 42:4 | 0,03023533 | 0,03785362 | 0,03366331 | 0,00054222 | 0 | 0 |
| PC 42:5 | 0,07226678 | 0,08440627 | 0,07205187 | 0,01514601 | 0,01342824 | 0,00786198 |
| PC 42:6 | 0,32920812 | 0,23633016 | 0,21946634 | 0,00582306 | 0,0177256 | 0,0364073 |
| PC 42:7 | 0,07841434 | 0,05157133 | 0,04547775 | 0 | 0,0019545 | 0,00101668 |
| PC 42:8 | 0,02113001 | 0,0191263 | 0,01426847 | 0 | 0 | 0 |
| PC 42:9 | 0,06812628 | 0,03339869 | 0,06172528 | 0,00724281 | 0,02356689 | 0,03413548 |
| PC 44:2 | 0,12636328 | 0,19272487 | 0,13551513 | 0,05555971 | 0,06076461 | 0,06307252 |
| PC 44:3 | 0,032503 | 0,04821414 | 0,03608396 | 0 | 0 | 0 |
| PC 44:6 | 0,06656611 | 0,07290048 | 0,06021198 | 0 | 0 | 0 |
| PC 44:7 | 0,01722669 | 0,00532353 | 0,00418505 | 0 | 0 | 0,00044063 |
| PC 44:9 | 0,05469644 | 0,06537268 | 0,05255189 | 0,00486481 | 0,00889552 | 0,0088154 |
| PC O-30:0 | 0,00122906 | 0,00049151 | 0,00087325 | 0,01002437 | 0,01639416 | 0,02350979 |
| PC O-30:1 | 0,00734711 | 0,00396304 | 0,00493868 | 0,01858054 | 0,01845854 | 0,02654127 |
| PC O-32:0 | 0,00108575 | 0,0022699 | 0,0025234 | 0,10710082 | 0,13378124 | 0,11567566 |
| PC O-32:1 | 0,03300971 | 0,03694669 | 0,03011615 | 0,15895301 | 0,18920032 | 0,18044517 |
| PC O-32:2 | 0,03215423 | 0,01953789 | 0,02422421 | 0,01227506 | 0,01993399 | 0,0376016 |
| PC O-34:0 | 0 | 0 | 0 | 0,0083715 | 0,0344827 | 0,02580789 |
| PC O-34:1 | 0,04624065 | 0,04287259 | 0,0426913 | 0,28645772 | 0,36938874 | 0,3515557 |
| PC O-34:2 | 0,01278128 | 0,02533338 | 0,00818932 | 0,14205675 | 0,18496028 | 0,1993069 |
| PC O-36:1 | 0 | 0 | 0 | 0,00831163 | 0,04027829 | 0,03402182 |
| PC O-36:2 | 0,05022583 | 0,0491466 | 0,04640771 | 0,04384555 | 0,0793786 | 0,07203004 |
| PC O-36:3 | 0,01012175 | 0,00667953 | 0,00725203 | 0,00309001 | 0,02391344 | 0,02700095 |
| PC O-36:4 | 0,01722264 | 0,00253677 | 0,0044177 | 0,01016293 | 0,03720847 | 0,04345722 |
| PC O-36:5 | 0,01426321 | 0,00401443 | 0,00599164 | 0,0046752 | 0,02980781 | 0,04296146 |
| PC O-38:1 | 0,01600138 | 0,02367461 | 0,01763838 | 0,03442898 | 0,0615435 | 0,05656966 |

| | | | | | | |
|---|---|---|---|---|---|---|
| PC O-38:2 | 0,00081772 | 0,00187222 | 0,00073808 | 0 | 0,00556478 | 0,0054364 |
| PC O-38:4 | 0 | 0 | 0 | 0 | 0,00814863 | 0,01457218 |
| PC O-38:6 | 0,01144798 | 0,001058 | 0,00166223 | 0 | 0,01175844 | 0,02932208 |
| PC O-40:1 | 0,00506544 | 0,0136993 | 0,00926231 | 0,04088381 | 0,05592994 | 0,05696048 |
| PC O-40:5 | 0 | 0,00094262 | 0,00029045 | 0 | 0,00930347 | 0,02519148 |
| PC O-40:6 | 0,00121713 | 0 | 0 | 0 | 0,01774616 | 0,02831603 |
| PC O-40:7 | 0,00144588 | 0,00066883 | 0,00068806 | 0 | 0,02309334 | 0,03707925 |
| PC O-42:5 | 0 | 0,00320354 | 0 | 0,03010486 | 0,04736349 | 0,04445832 |
| PC O-42:6 | 0,0013231 | 0,00022538 | 0,00031449 | 0 | 0,01001694 | 0,04517889 |
| PC O-44:4 | 0,02969025 | 0,09732741 | 0,05154247 | 0,00067766 | 0,01534913 | 0,02450945 |
| PC O-44:5 | 0,00020585 | 0,00038385 | 0,00028459 | 0,0059365 | 0,01034842 | 0,01383015 |
| PE 32:1 | 0,19610711 | 0,19297884 | 0,29025194 | 0,01204906 | 0,01113481 | 0,05171128 |
| PE 34:1 | 0,61463609 | 0,65455623 | 1,20095444 | 0,16535178 | 0,19668178 | 0,25038626 |
| PE 34:2 | 1,03995192 | 0,86981162 | 1,40066044 | 0,34008032 | 0,33785144 | 0,41736543 |
| PE 34:3 | 0,05594799 | 0,06423378 | 0,12935383 | 0 | 0 | 0 |
| PE 36:1 | 0,45814596 | 0,43494141 | 0,92567345 | 0,02957603 | 0,04757926 | 0,12994451 |
| PE 36:2 | 2,32973518 | 2,21048308 | 3,99175982 | 0,53653019 | 0,65005686 | 0,79851577 |
| PE 36:3 | 0,23470018 | 0,20745473 | 0,36128454 | 0,00239355 | 0,01167197 | 0,0339381 |
| PE 36:4 | 0,16179769 | 0,13571329 | 0,23497703 | 0,00523478 | 0,00590677 | 0,01999259 |
| PE 36:5 | 0,0491753 | 0,01328539 | 0,05303202 | 0 | 0 | 0,00043598 |
| PE 38:2 | 0,15528567 | 0,15094476 | 0,3305465 | 0,00244385 | 0,00364171 | 0,02991123 |
| PE 38:3 | 0,07993726 | 0,05206002 | 0,15026116 | 0 | 0,00340669 | 0,00375334 |
| PE 38:4 | 0,64512751 | 0,62570242 | 1,14233275 | 0,02053588 | 0,01557345 | 0,08322949 |
| PE 38:5 | 0,61543569 | 0,52524154 | 0,85117299 | 0,00999882 | 0,03241585 | 0,1410662 |
| PE 38:6 | 0,13493766 | 0,07267793 | 0,17068744 | 0 | 0 | 0 |
| PE 40:2 | 0,00494923 | 0,00308547 | 0,01055069 | 0 | 0 | 0 |
| PE 40:5 | 0,1470829 | 0,14608025 | 0,28974549 | 0 | 0 | 0,00321994 |
| PE 40:6 | 0,32122448 | 0,25793424 | 0,48046783 | 0 | 0 | 0 |
| PE 40:7 | 0,13506342 | 0,10898017 | 0,19007728 | 0 | 0,00210369 | 0,00287862 |
| PG 32:1 | 0,00163516 | 0,00406546 | 0,00397868 | 0,02932222 | 0,04842408 | 0,05511632 |
| PG 32:2 | 0,00053155 | 0,00046188 | 0,00170746 | 0,02107182 | 0,03111214 | 0,03710692 |
| PG 34:1 | 0,0691465 | 0,07539904 | 0,08064074 | 0,10419016 | 0,09404343 | 0,12031095 |

| | | | | | | |
|---|---|---|---|---|---|---|
| PG 34:2 | 0,02397721 | 0,02113474 | 0,02388413 | 0,41461267 | 0,42548929 | 0,45265084 |
| PG 34:3 | 0 | 0 | 0 | 0,03616821 | 0,04274106 | 0,05100111 |
| PG 36:2 | 0,05466789 | 0,03821419 | 0,04758855 | 0,97199208 | 0,89562495 | 1,12919175 |
| PG 36:3 | 0,00559572 | 0,00068174 | 0,00238195 | 0,20097849 | 0,19787276 | 0,23060434 |
| PG 36:4 | 0,00021465 | 0,0003179 | 0 | 0,06878159 | 0,07884792 | 0,08449785 |
| PG 36:5 | 0 | 0 | 0,00016825 | 0,00764117 | 0,0149845 | 0,02827447 |
| PG 38:3 | 0,00010773 | 0 | 0 | 0,02188955 | 0,03307547 | 0,04965287 |
| PG 38:4 | 0,00413883 | 0,00020416 | 0,00093232 | 0,15480154 | 0,17195989 | 0,21219141 |
| PG 38:5 | 0,00102503 | 0 | 0 | 0,13089918 | 0,13315339 | 0,16321605 |
| PG 38:6 | 0,00281157 | 0,00046989 | 0,00054772 | 0,13062591 | 0,13617965 | 0,15447201 |
| PG 38:7 | 0,00211171 | 0,00020958 | 0,00059707 | 0,18026811 | 0,17145432 | 0,2005745 |
| PG 40:5 | 0 | 0 | 0 | 0,00833613 | 0,01404693 | 0,04528862 |
| PG 40:6 | 0,00059453 | 0 | 0 | 0,11506063 | 0,10094052 | 0,13812689 |
| PG 40:7 | 0,04560513 | 0,0237042 | 0,03146363 | 0,94828115 | 0,91787512 | 1,10704258 |
| PG 40:8 | 0,00135893 | 0 | 0 | 0,11015243 | 0,09847424 | 0,11891641 |
| PG 42:10 | 0,00019891 | 0 | 0 | 0,06407446 | 0,06821847 | 0,08422938 |
| PG 42:7 | 0 | 0 | 0 | 0,00480264 | 0 | 0,01282847 |
| PG 42:8 | 0 | 0 | 0 | 0,0050477 | 0,00964245 | 0,0358981 |
| PG 42:9 | 0,00165253 | 0 | 0,00043721 | 0,11728795 | 0,11730995 | 0,13107939 |
| PG 44:10 | 0 | 0 | 0 | 0 | 0,00160007 | 0,02452567 |
| PG 44:11 | 0,00096022 | 0,00030418 | 0,00018851 | 0,07963232 | 0,06442082 | 0,09204498 |
| PG 44:12 | 0,01576679 | 0,00447495 | 0,00469847 | 0,41234634 | 0,35699269 | 0,40750338 |
| PI 30:0 | 0 | 0 | 0 | 0 | 0 | 0 |
| PI 30:1 | 0,00103036 | 0,0084426 | 0,00711998 | 0 | 0 | 0 |
| PI 32:0 | 0,04251301 | 0,12317113 | 0,11238451 | 0,04654746 | 0,05072234 | 0,10307142 |
| PI 32:1 | 0,20519187 | 0,29714355 | 0,27353346 | 0,19990508 | 0,21512625 | 0,29787388 |
| PI 34:1 | 0,93528034 | 1,34069865 | 1,33711685 | 1,01943011 | 0,89873137 | 1,24186897 |
| PI 34:2 | 0,44987963 | 0,62859749 | 0,60978438 | 0,33776718 | 0,3995498 | 0,51837751 |
| PI 36:1 | 0,63733505 | 0,86992777 | 0,90809047 | 0,66649981 | 0,55616056 | 0,65727017 |
| PI 36:2 | 0,7668915 | 1,0498636 | 1,06143841 | 0,4524081 | 0,50669734 | 0,75844459 |
| PI 36:3 | 0,09793751 | 0,11422945 | 0,12471781 | 0,00950894 | 0,00846987 | 0,05485178 |
| PI 36:4 | 0,07805631 | 0,09443388 | 0,0762891 | 0,01303038 | 0,01378122 | 0,05228865 |

| | | | | | | |
|---|---|---|---|---|---|---|
| PI 36:5 | 0 | 0 | 0 | 0 | 0 | 0 |
| PI 38:1 | 0,03276337 | 0,02065379 | 0,0613901 | 0,00163907 | 0,0013621 | 0,0102872 |
| PI 38:2 | 0,07764388 | 0,11035229 | 0,12180931 | 0,00472005 | 0,01065432 | 0,02558567 |
| PI 38:3 | 0,11623889 | 0,13957267 | 0,15147677 | 0,02669096 | 0,00818636 | 0,08310624 |
| PI 38:4 | 0,23903348 | 0,24592412 | 0,24398773 | 0,08678158 | 0,10269856 | 0,20785773 |
| PI 38:5 | 0,15621615 | 0,17212663 | 0,16124065 | 0,01278557 | 0,03393061 | 0,11731704 |
| PI 38:6 | 0,1046091 | 0,13673989 | 0,1369594 | 0,00432012 | 0,01406094 | 0,07034689 |
| PI 40:3 | 0,16369966 | 0,22078767 | 0,1995903 | 0,01177575 | 0,06242321 | 0,09808946 |
| PI 40:4 | 0,03329699 | 0,02144048 | 0,03748467 | 0 | 0 | 0 |
| PI 40:5 | 0,10199443 | 0,12131762 | 0,13057759 | 0,00234522 | 0,00183336 | 0,04585316 |
| PI 40:6 | 0,19388783 | 0,23227668 | 0,2458986 | 0,03296525 | 0,04834414 | 0,14248615 |
| PI 40:7 | 0,08597882 | 0,11635506 | 0,11609713 | 0,0058932 | 0,00167661 | 0,03676523 |
| PI 42:5 | 0,00634123 | 0,00070455 | 0,01085036 | 0 | 0 | 0 |
| PI 42:6 | 0,00335663 | 0,0007573 | 0,00098576 | 0 | 0 | 0 |
| PS 32:1 | 0,03402065 | 0,05865031 | 0,05161113 | 0,01705341 | 0,05013761 | 0,06554282 |
| PS 34:1 | 0,19952746 | 0,27103762 | 0,27509045 | 0,26056979 | 0,27789688 | 0,25092632 |
| PS 34:2 | 0,09323669 | 0,10760498 | 0,11895442 | 0,06753208 | 0,08867687 | 0,13464437 |
| PS 36:1 | 0,24179286 | 0,32225381 | 0,34629556 | 0,49373383 | 0,40192521 | 0,40454717 |
| PS 36:2 | 0,22503827 | 0,26858159 | 0,29901005 | 0,28660963 | 0,23284865 | 0,29406186 |
| PS 36:4 | 0,01996431 | 0,02567176 | 0,01408427 | 0,00409079 | 0,00984374 | 0,0176818 |
| PS 38:2 | 0 | 0 | 0 | 0 | 0 | 0 |
| PS 38:4 | 0,04280022 | 0,05897063 | 0,04980501 | 0,06389125 | 0,07924201 | 0,07067697 |
| PS 40:2 | 0 | 0 | 0 | 0 | 0 | 0 |
| PS 40:6 | 0,01377818 | 0,01905776 | 0,01486132 | 0,03595287 | 0,03180262 | 0,0290795 |
| PS 40:7 | 0,01336307 | 0,02139324 | 0,0237179 | 0,00112388 | 0,01015172 | 0,01911665 |
| PS 42:7 | 0 | 0 | 0 | 0 | 0 | 0 |
| SM 32:0;2 | 0,0015936 | 0 | 0 | 0,00193833 | 0,00131749 | 0,00353658 |
| SM 32:1;2 | 0,10745281 | 0,08833946 | 0,08348543 | 0,32348216 | 0,21658843 | 0,23860145 |
| SM 32:2;2 | 0 | 0 | 0 | 0 | 0 | 0 |
| SM 34:0;2 | 0,15932228 | 0,13067441 | 0,12613152 | 0,4811948 | 0,32492569 | 0,29689865 |
| SM 34:1;2 | 1,64314177 | 1,14631095 | 1,24382277 | 5,12327113 | 3,78648571 | 3,97256943 |
| SM 34:2;2 | 0,14994064 | 0,12226572 | 0,12147194 | 0,45978412 | 0,34281541 | 0,36686078 |

| | | | | | | |
|---|---|---|---|---|---|---|
| SM 36:0;2 | 0 | 0 | 0 | 0 | 0,0014329 | 0 |
| SM 36:1;2 | 0,04086106 | 0,05931999 | 0,05091247 | 0,23268828 | 0,35791041 | 0,31467479 |
| SM 36:2;2 | 0,00655044 | 0,00128719 | 0,00254157 | 0,04653909 | 0,08944349 | 0,11396221 |
| SM 38:1;2 | 0 | 0,00263131 | 0 | 0,05388621 | 0,15532852 | 0,1359126 |
| SM 38:2;2 | 0 | 0 | 0 | 0 | 0,00312415 | 0,02681886 |
| SM 40:0;2 | 0 | 0 | 0 | 0 | 0,00332252 | 0,0060237 |
| SM 40:1;2 | 0 | 0,14239327 | 0,0781725 | 0,33389729 | 0,60441019 | 0,53896224 |
| SM 40:2;2 | 0,050607 | 0,0583611 | 0,04813198 | 0,16115756 | 0,26543896 | 0,23898872 |
| SM 42:1;2 | 0,08479841 | 0,16420226 | 0,09294348 | 0,22754902 | 0,36216754 | 0,35116829 |
| SM 42:2;2 | 0,74382337 | 0,90393666 | 0,69603109 | 1,4535621 | 1,72360496 | 1,5003932 |
| SM 42:3;2 | 0,11977836 | 0,11441482 | 0,10252989 | 0,15251053 | 0,214133 | 0,21488659 |
| SM 44:2;2 | 0,08448847 | 0,10719905 | 0,07743757 | 0,12486314 | 0,12406316 | 0,1164994 |
