# Supplementary material for: Galactosyl- and glucosylsphingosine induce lysosomal membrane permeabilization and cell death in cancer cells
Source: PLoS One. 2022 Nov 21;17(11):e0277058. doi: 10.1371/journal.pone.0277058 (PMC9678304; doi:10.1371/journal.pone.0277058)

Raw images of western blots in figure 1D

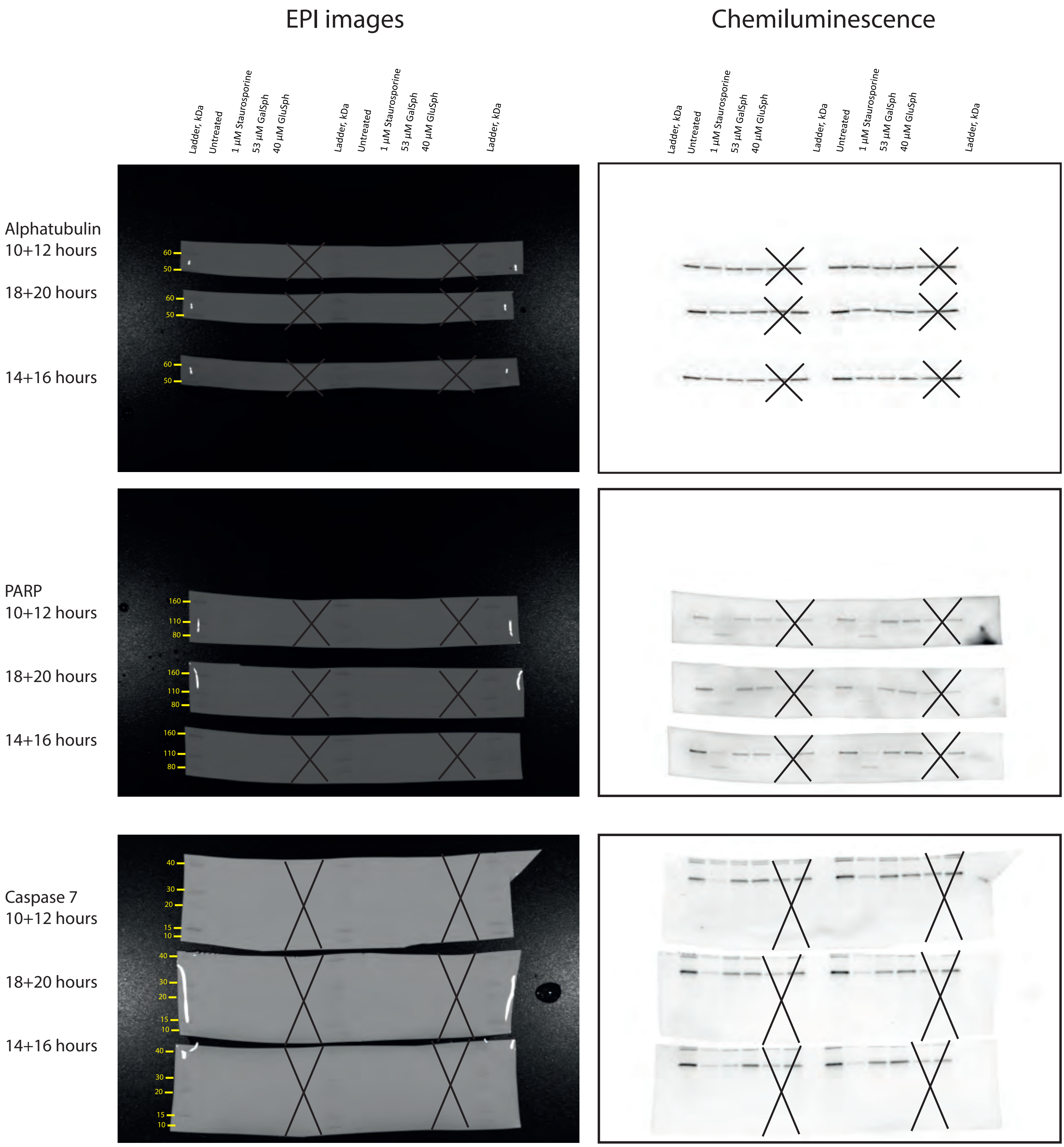

Raw images of western blots in figure 5A

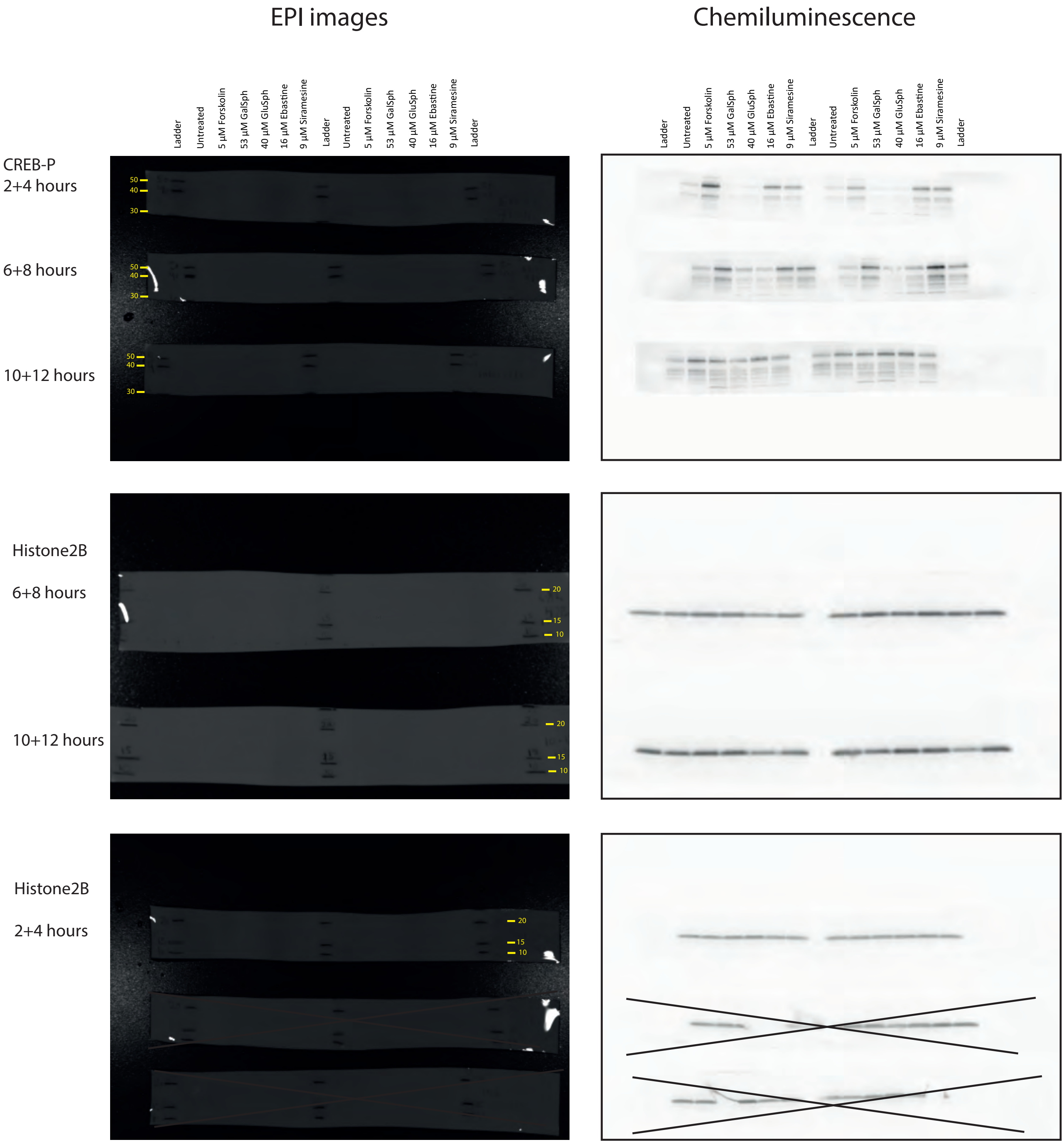

Raw images of western blots in figure 5F

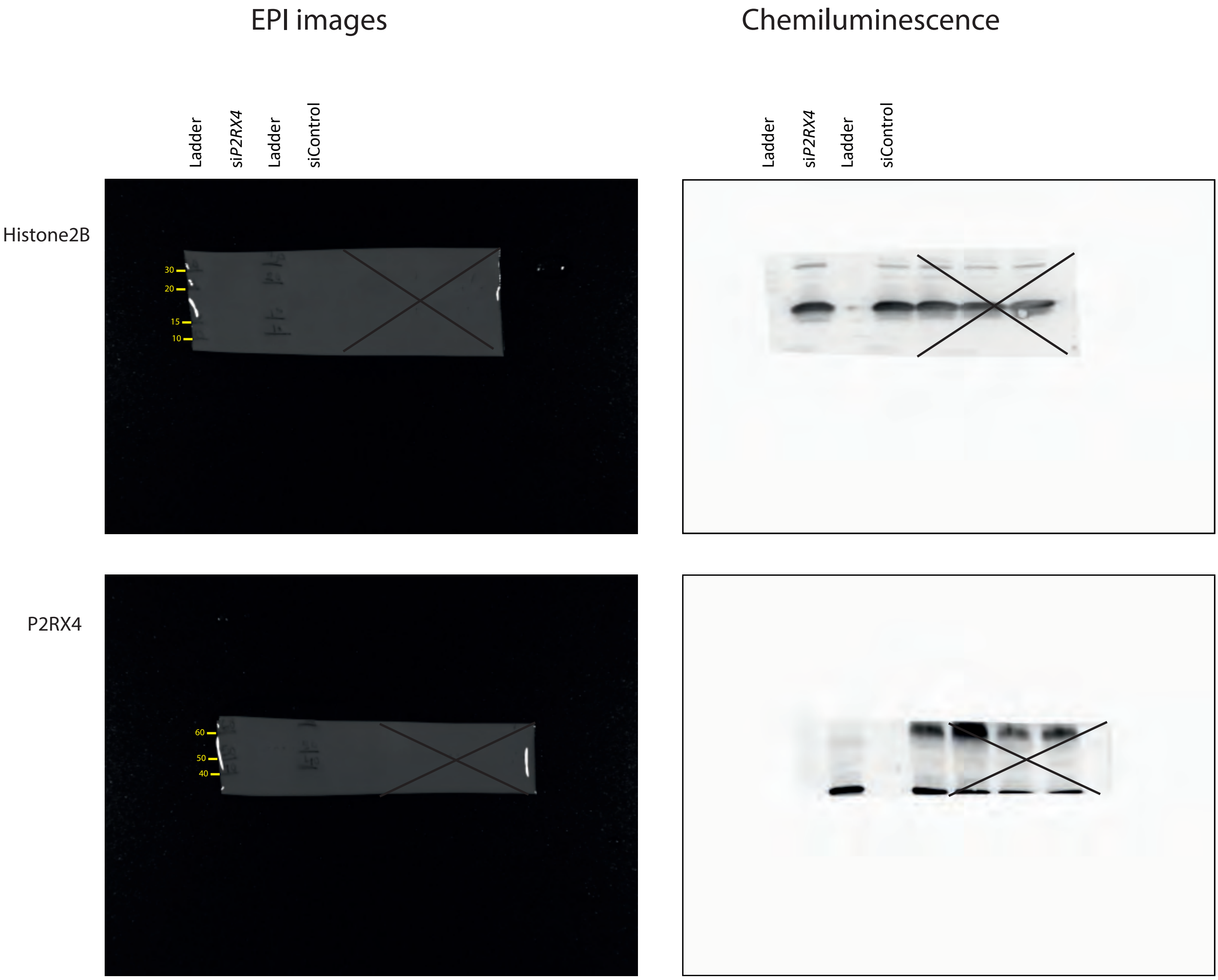

Supplement: S1 File — (PDF) [file pone.0277058.s009.pdf]
