# Supplementary material for: Galactosyl- and glucosylsphingosine induce lysosomal membrane permeabilization and cell death in cancer cells
Source: PLoS One. 2022 Nov 21;17(11):e0277058. doi: 10.1371/journal.pone.0277058 (PMC9678304; doi:10.1371/journal.pone.0277058)
Supplement: Attachment — Submitted filename: RESPON~1.DOC [file pone.0277058.s011.DOC]

Comment from Reviewer 3

In the revised version, the authors have done some additional experiments and added some explanations about them. However, the interpretation of some results is not convincing and one of the required experiments has not been done. Therefore, the authors' efforts to improve the manuscript are recognized, but not sufficient.

Specific comments:

[Original comment #2] 
Figure 5: It is desirable to analyze intracellular cAMP levels and Ca2+ efflux, as the authors did in a previous paper. In addition, it is considered that the involvement of cAMP can be clarified by using lysophospholipids in combination with a reagent that lowers cAMP rather than in combination with forskolin that raises cAMP. Similarly, it is desirable to analyze the lysosomal pH.

[Authors’ response #2]
We have previously measured Ca2+ release after treatment with the lysosphingolipids, but the result has been inconclusive. We have also shown in the originally submitted manuscript that the knockdown of P2RX4 did not have any impact on the lysosphingolipid-induced cell death, and we therefore believe that lysosphingolipids does not induce rapid Ca2+ release like CADs. Our data suggest that lysosphingolipids and CADs induce similar manners of cell death, but they don’t induce cell death or lysosomal membrane permeabilization through the exact same mechanisms. Page 8 of 8 We have performed pH measurements, as suggested, to examine whether lysosphingolipids also neutralize lysosomes similarly to CADs prior to lysosomal membrane permeabilization. The lysosphingolipids did not neutralize the lysosome pH after one or five hours of treatments, despite the lysosphingolipids being weak bases like CADs. This new data is presented as a supplementary figure, S1 Fig and the results are described in lines 361-365, page 16: “Unlike CADs (50), the lysosphingolipids did not induce notable elevation in the lysosomal pH in MCF7 cells after one or five hours of treatments and thus prior to lysosomal leakage, when assessed after loading lysosomes with dextran coupled with pH-sensitive fluorescein and pH-insensitive tetramethylrhodamine (S1 Fig).”

[New comments on the authors' response #2]
In panel A, cAMP-dependent CREB phosphorylation was detected in 53 µM GalSph and 40 µM GlcSph, not analyzed in 40 µM GalSph and 28 µM GlcSph. On the other hand, in panel B, the effect of Forskolin on cell death was observed in 40 µM GalSph and 28 µM GlcSph, but not in 43-45 µM GalSph and 30-32 µM GlcSph. From this result, it is presumed that Forskolin does not enhance cell death even at 53 µM GalSph and 40 µM GlcSph. Therefore, from these results, it cannot be determined whether cAMP signaling is involved in the induction of cell death by GalSph or GlcSph. Again, it is thought that the involvement of cAMP can be clarified by using GalSph and GlcSph together with reagents that lower cAMP instead of Forskolin, which raises cAMP. This experiment is easy to try. Additionally, in panel C, the authors also performed P2RX4 knockdown experiments and showed that the induction of cell death by GalSph or GlcSph was not mediated by P2RX4. This experiment showed that the induction of cell death by GalSph or GlcSph was not dependent on the Ca2+ release through P2RX4, but not denied that the Ca2+ release through other channels was involved. Therefore, it is desirable to present the measurement of Ca2+ release as conclusive data. The authors need to explain in more detail, at least not to mislead the reader, even if they do not perform this experiment.

**Reply to reviewer’s comment (to ‘New comments on the authors’ response #2’)**

**We thank the reviewer for constructively commenting on our revised manuscript. We found the comment useful and fully agree. In the previous version of the manuscript, the discussion of presented data regarding the possible involvement of cAMP signalling pathway in the mechanism of cell death induced by lysosphingolipids indeed focused solely on the positive outcome achieved only under one of the tested conditions. We now have extended the discussion on the matter to also include the negative outcome, and most importantly, we write clearly that this matter still remains to be studied.**

**We have demonstrated in this manuscript that extracellularly supplied lysosphingolipids can destabilize the lysosomal membranes, similarly to cationic and amphiphilic drugs (CADs). At the same time, the lysosphingolipid-induced cell death is not dependent on Ca2+ release from P2RX4 unlike CADs, and the lysosphingolipids and CADs thus do not induce the same responses of signalling leading to the eventual cell death. We believe that properly revealing the signalling pathways induced by lysosphingolipids would be both challenging and labour-intensive, even though we agree with the reviewer that a few additional simple experiments might answer some of these questions e.g. involvement of cAMP. We therefore find it more suitable to further explore the signalling pathways of lysosphingolipids in other ongoing projects of the lab, and leave the questions not fully answered for the present manuscript.**

**We have expanded the discussion section with the following paragraph, in page 24, lines 557-570. Newly added sentences are marked with bold letters;**

*“Supporting this idea, we show here that CAD-resistant MCF7 cells are also resistant to the lysosphingolipid-induced cytotoxicity. Additionally, lysosphingolipids activate the cAMP signalling pathway and induce lysosomal leakage as early events occurring prior to cell death, in a manner similar to that observed in CAD-treated cells (22, 28).* ***The role of cAMP in lysosphingolipid-induced cell death is supported by the ability of forskolin to enhance cell death induced by lysosphingolipids at concentrations below their respective IC50 values. On the contrary, forskolin failed to enhance cell death when combined with higher concentrations of lysosphingolipids at around their respective IC50 values, which were used here to demonstrate the phosphorylation of CREB.*** *In addition, the early lysosphingolipid-induced signalling pathway leading to the cAMP accumulation and cell death in MCF7 cells appears different from the P2RX4- and Ca^2+^-dependent pathway induced by CADs because the depletion of P2RX4 in MCF7 cells had no effect on the lysosphingolipid-induced cytotoxicity.* ***Thus, how lysosphingolipids activate the cAMP signalling pathway and whether it is involved in the cytotoxic mechanism of lysosphingolipids remain to be studied.****”*
